# Impact of weight‐loss interventions on psoriasis severity: A systematic review and meta‐analysis

**DOI:** 10.1111/jdv.70247

**Published:** 2025-12-19

**Authors:** Sarah Morrow, Poppy Hawkins, Christopher E. M. Griffiths, Thanasis G. Tektonidis, Eli Harriss, Jadine Scragg, Susan Jebb

**Affiliations:** ^1^ University of Oxford Oxford UK; ^2^ University of Hertfordshire Hertfordshire UK; ^3^ King's College London London UK; ^4^ Oxford Brookes University Oxford UK

**Keywords:** diet, exercise, glucagon‐like peptide‐1 receptor agonist, medical dermatology, PASI, physical activity, psoriasis, quality‐of‐life, weight

## Abstract

**Background:**

Psoriasis affects at least 60 million people worldwide, and 80% also live with overweight or obesity. Excess weight increases susceptibility to psoriasis and is associated with more severe disease.

**Objectives:**

To evaluate the impact of weight‐loss interventions on psoriasis severity (Psoriasis Area and Severity Index [PASI], PASI50, PASI75, PASI100 [50%/75%/100% reduction in baseline PASI, respectively]) and quality of life (Dermatology Life Quality Index [DLQI]).

**Methods:**

We systematically searched five databases and two trial registries (inception to 03/09/2025). Outcomes were informed by patient focus‐group discussions. Randomized controlled trials (RCTs) in adults with psoriasis, comparing any weight‐loss intervention versus usual care or a lower‐intensity weight‐loss intervention, were included. Studies had to report a change in weight and ≥1 psoriasis severity or quality‐of‐life measure. Random effects meta‐analyses were used.

**Results:**

Thirteen RCTs (1145 participants) with 14 comparisons were included. Eleven interventions advised dietary changes, of which four included physical activity. Three used weight‐loss medications. Across 14 comparisons (*n* = 1145, mean difference (MD) in weight change: −6.7 kg), weight‐loss interventions produced a greater reduction in PASI versus control: MD −2.5 (95%CI: −3.8 to −1.1, *I* ^2^ = 85.2%). We found a significant effect of weight‐loss interventions on the likelihood of achieving PASI75 (RR = 1.6, 95%CI: 1.1–2.2, *I* ^2^ = 22.6% [based on six comparisons, *n* = 681, MD in weight change: −7.3 kg]). There was no statistically significant effect of the interventions on the likelihood of achieving PASI50 (RR = 1.5, 95%CI: 0.9–2.4, *I* ^2^ = 72.8% [based on four comparisons, *n* = 509, MD in weight change: −4.0 kg]) or PASI100 (RR = 1.6, 95%CI: 0.3–9.7, *I* ^2^ = 0.0% [based on two comparisons, *n* = 334, MD in weight change: −5.2 kg]), but both analyses were limited by few studies. Across seven comparisons (*n* = 364; MD in weight change −7.8 kg), weight‐loss interventions were associated with a significant improvement in DLQI compared to control: MD −5.0 (95%CI: −9.7 to −0.3, *I* ^2^ = 96.0%).

**Conclusions:**

High‐certainty evidence suggests weight‐loss interventions can improve psoriasis severity and quality of life, and should be considered as part of routine treatment.

## Graphical abstract

Weight‐loss interventions were associated with clinically and statistically significant improvements in psoriasis severity (PASI and PASI 75) and quality of life (DLQI). These findings support the role of weight‐loss interventions as effective adjuncts to medical therapy and their integration into holistic psoriasis care.

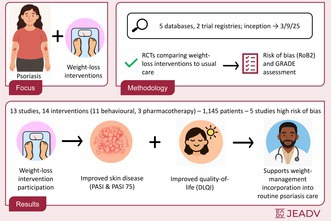

**Why was the study undertaken?**

People with psoriasis increasingly ask how behavioural or lifestyle changes might improve their skin disease. Weight management may improve overall health and may have benefits for the skin. Reducing psoriasis severity can improve quality of life, a top priority for patients. Previous meta‐analyses only included behavioural weight‐loss interventions and did not explore quality‐of‐life. We addressed these gaps by evaluating the impact of any weight‐loss intervention ‐ behavioural or pharmacological ‐ on psoriasis severity and quality‐of‐life outcomes.

**What does this study add?**

Weight‐loss interventions, including both behavioural and pharmacological approaches, were associated with clinically and statistically significant improvements in psoriasis severity (PASI and PASI 75) and quality‐of‐life (DLQI). Data were limited and there was considerable heterogeneity. The certainty of evidence was high for PASI and DLQI, moderate for PASI 75 and PASI 50, and low for PASI 100.

**What are the implications of this study for disease understanding and/or clinical care?**

Our patient advisory group helped shape the outcome selection and result interpretation, confirming the findings as meaningful and motivating from a lived experience perspective. Patients felt reassured that the demands of weight‐loss programmes nonetheless improved their quality of life. These findings support the integration of weight‐loss interventions, as an adjunct to medical care, into routine psoriasis management and highlight the importance of addressing lifestyle alongside more traditional treatment modalities.

## INTRODUCTION

Psoriasis affects at least 60 million people worldwide, significantly impairing quality of life.^1^, ^2^, ^3^, ^4^ People with severe psoriasis are more than twice as likely to have obesity compared to those without psoriasis (odds ratio [OR] 2.23).^5^, ^6^ Similarly, higher body mass index (BMI) correlates with increased psoriasis severity and worsens quality of life.^7^, ^8^, ^9^, ^10^

Excess weight reduces the efficacy of systemic therapy and limits treatment options.^11^, ^12^, ^13^, ^14^, ^15^, ^16^ Obesity‐related conditions can contraindicate certain medications routinely used to treat psoriasis and increase the complications of others, such as acitretin and methotrexate.^5^, ^17^ Weight‐loss interventions might reduce psoriasis severity by dampening systemic inflammation, improving insulin resistance and/or reducing inflammation through the psoriasis‐metabolic‐syndrome axis.^18^ Clinical guidelines recommend behavioural weight management support for patients living with psoriasis.^19^ However, dermatology clinicians often hesitate to address weight due to concerns about patient rapport, time constraints and limited expertise.^20^, ^21^, ^22^

Previous systematic reviews show that behavioural weight‐loss interventions, typically low‐energy diets, result in greater weight loss and improvements in psoriasis area and severity index (PASI) than standard care for people with psoriasis.^19^, ^23^ However, no reviews have considered the full range of weight‐loss interventions, such as surgery and pharmacotherapy, or had insufficient data to meta‐analyse important patient‐reported outcomes such as dermatology life quality index (DLQI).

This systematic review and meta‐analysis aimed to address this evidence gap by synthesising and quantifying the impact of any weight‐loss intervention on psoriasis severity and/or patient quality of life.

## METHODS

This review was prospectively registered (PROSPERO 2023 CRD42023485378) and conducted per the *Cochrane Handbook for Systematic Reviews of Interventions* ^24^ and Preferred Reporting Items for Systematic Reviews and Meta‐Analyses (PRISMA) reporting guidelines.^25^

### Search strategy

Ovid MEDLINE, Ovid Embase, Ovid PsycINFO, EBSCOhost CINAHL, Cochrane Central Register of Controlled Trials, Web of Science Core Collection and clinicaltrials.gov were searched from inception to 03/09/2025 by an information specialist (EH), using the Cochrane RCT filter.^26^ Search terms were informed by relevant systematic reviews.^19^, ^23^, ^27^, ^28^, ^29^ No language or country restrictions were applied (full search strategy: Appendix S1).

Relevant systematic reviews identified were moved to full‐text review for additional reference screening. Unavailable papers were requested from authors.

### Inclusion criteria

Included randomized controlled trials (RCTs) enrolled adults (≥18 years) with psoriasis and assessed weight‐loss interventions (behavioural [diet and/or exercise], pharmacological or surgical) on psoriasis severity. Interventions were required to be known to induce weight loss. Pharmacological agents had to be currently or previously licensed for weight loss or share a class effect with a licensed agent.

Studies required a comparator group receiving usual psoriasis care or a lower‐intensity weight‐loss intervention. We considered minimal interventions, such as advice‐only, to fall under ‘usual care’, based on current psoriasis clinical guidelines.^30^ Intervention intensity was assessed based on behavioural support and energy deficit.^29^, ^31^ Studies comparing interventions of the same intensity were excluded.

Studies had to report at least one measure of weight change (e.g. BMI) and one measure of psoriasis severity—such as change in PASI or the proportion of participants achieving a 50%, 75% or 100% reduction in PASI from baseline (PASI50/75/100)—or a quality‐of‐life measure (e.g. DLQI). No restrictions were placed on trial duration, follow‐up or language.

### Data collection and quality assessment

Studies were independently screened by pairs of researchers, and data were extracted using Covidence.^32^ Discrepancies were resolved through discussion, and authors were contacted for clarifications or missing data. Weight change was treated as a process measure, as weight loss interventions vary in effectiveness. When multiple analyses were available, intention‐to‐treat analyses were extracted as the most conservative.

Two independent reviewers assessed the risk of bias using the Cochrane RoB2 tool. As blinding of participants and personnel is not feasible for most behavioural interventions, we considered whether the lack of blinding in each study was likely to have led to systematic deviations from the intended intervention, following RoB2 guidance and precedent.^33^ Given the typically high attrition in weight‐loss trials, we followed Cochrane guidance to assess bias from missing outcome data, considering the proportion missing, group differences and reasons for loss‐to‐follow‐up. Since PASI scoring relies on physician judgement, studies without blinded assessors were rated high risk for outcome measurement. Funnel plots assessed publication bias and small‐study effects when ≥10 studies were available.^34^

### Data synthesis and analysis

We estimated outcome changes from baseline to intervention end for all relevant trial groups. Mean differences in outcomes between groups and corresponding standard deviations (SD) were extracted per *Cochrane* guidelines.^35^ Full details on data extraction and handling are available (Table S1).

Meta‐analyses were performed in Stata SE (v18.5) for outcomes with ≥2 studies, comparing psoriasis severity changes between intervention and control arms. Pooled results were expressed as mean differences (95% confidence intervals [CI]) for PASI and DLQI score changes. PASI50/75/100 proportions were analysed using inverse‐variance‐weighted risk ratios (RR), where RR > 1.0 favoured the intervention and RR < 1.0 favoured the control.

Meta‐analyses used a random effects model (Hartung–Knapp–Sidik–Jonkman, HKSJ) to account for methodological and clinical heterogeneity, providing less biased effect estimates and 95%CI.^36^ Heterogeneity was assessed using the *I* ^2^ statistic.^37^

Studies differed in their data imputation methods for participants lost to follow‐up. We analysed the data as reported. In multi‐arm studies, the control group sample size was proportionally split between comparisons to prevent double‐counting. Precision was indicated by narrower confidence intervals and consistency by alignment in the direction of effect estimates across studies. Certainty of evidence was evaluated using GRADE (Grading of Recommendations Assessment, Development and Evaluation).^38^

### Sensitivity analyses

We conducted three prespecified sensitivity analyses to explore potential causes of heterogeneity, each excluding studies at high risk of bias. Post‐hoc sensitivity analyses excluded studies where weight loss was minimal, studies with mild baseline psoriasis (PASI < 5), the study in which psoriasis treatments differed between groups, and two studies that did not report a variance measure for change in PASI.

### Subgroup analyses

Subgroup analyses were conducted by intervention type (diet, diet+exercise and pharmacological), duration of intervention and mean participant baseline PASI to assess possible sources of heterogeneity.

### Presentation of results

Forest plots display data by mean difference in weight change, in descending order.

### Public involvement

Thirty‐two people with psoriasis helped prioritize outcomes.^39^ They wanted clear, scientific guidance on the impact of weight management on skin and wellbeing, leading us to prioritize quality of life as an outcome alongside psoriasis severity.

## RESULTS

**FIGURE 1:**
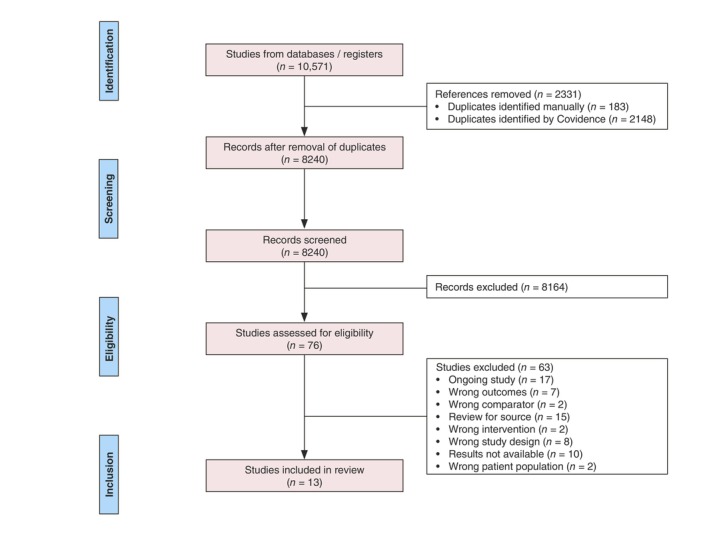
Preferred Reporting Items for Systematic Reviews and Meta‐Analyses (PRISMA) flowchart to demonstrate review process.

Of 10,571 identified articles, 13 studies (14 comparisons) were included (Figure 1).

### Characteristics of included studies

Tables 1 and 2 summarize study characteristics and key findings. Of 1145 randomized participants across the studies, follow‐up data were available for 1063. The reported methods for handling missing data in each study are detailed (Table S2).

**TABLE 1:** Characteristics of the included studies.

Study (Author, year)	Country	Population (BMI units = kg/m^2^)	Number of participants^a^ (intervention: control)	Age (*years*) (mean (SD))	Female participants (%)	Intervention	Control	Other medical treatments for psoriasis	Intervention duration (weeks)	Final follow‐up (weeks)
Al‐Mutairi (2014)	Kuwait	Overweight or obese (25 ≤ BMI < 35) Moderate–severe psoriasis	131:131	46.9 (6.4)	64.5	Caloric restriction (<1000 kcal/day)	No lifestyle changes	All participants: biologic therapy	8	24^b^
Faurschou (2015)	Denmark	Overweight or obese Moderate‐to‐severe psoriasis (PASI≥8)	11:9	*51.3 (13.0)*	25.0	Once‐daily liraglutide injections (starting 0.6 mg/day and increasing to 1.8 mg/day over first 3 weeks of intervention – then maintained)	Once‐daily placebo injections	No specific treatments, participants used a mixture of systemic and topical treatments	8	8
Gisondi (2008)	Italy	Obese (30 ≤ BMI < 40) Clinically stable plaque psoriasis (PASI ≥ 10, affecting ≥10% BSA)	30:31	*51.6 (12.5)*	50.8	Low‐calorie diet (daily kcal intake = 500 kcal below REE) Plus, moderate physical activity for 40 min, 4×/week	Moderate physical activity for 40 min, 4×/week	All participants: ciclosporin (2.5 mg/kg/day)	24	24 (52)^c^
Guida (2014)	Italy	Obese (BMI > 30) Stable, mild‐to‐severe chronic plaque psoriasis	22:22	*52.0 (10.9)*	27.3	Caloric restriction (20 kcal/kg/day) +Diet rich in n‐3 PUFAs	No lifestyle changes	All participants: systemic immunosuppressive therapy	26	26
Ismail (2023)	Egypt	Obese (class 1) (30 ≤ BMI < 35) Stable, mild‐to‐severe chronic plaque psoriasis Male NAFLD	32:32	*51.7 (11.3)*	0	Low‐calorie diet (daily kcal intake = 500 kcal below BMR) +15,000 steps/day walking programme	No lifestyle changes	All participants: systemic immunosuppressive therapy	12	12
Ismail (2024)	Egypt	Obese (class 1) (30 ≤ BMI < 35) Mild‐to‐severe chronic plaque psoriasis Male Erectile dysfunction Metabolic syndrome	30:30	*44.4 (3.4)*	0	Low‐calorie diet (daily kcal intake = 500 kcal below BMR) +Supervised treadmill walking programme for 40 min, 3×/week	No lifestyle changes	All participants: systemic immunosuppressive therapy	12	12
Jensen (2013)	Denmark	Overweight or obese (BMI > 27) Chronic plaque psoriasis	30:30	50.8 (10.3)	47.0	2‐staged dietary intervention First stage 800–1000 kcal/day, TDR Second stage ~1200 kcal/day, blended TDR +Group sessions	Conventional diet Same group sessions as intervention arm, reinforcing normal, healthy diet	No specific treatments, participants used a mixture of systemic and topical treatments	16	16 (64)^d^
Kimball (2012)	USA	Overweight or obese (BMI > 25) Chronic moderate‐to‐severe plaque psoriasis (PASI≥10)	10 (OD):10 (SB):10 (control)	*47.2 (13.8)*	36.7	OD: low‐fat, vegetarian diet SB: 3 phases: low‐carbohydrate, low‐GI, maintenance	No lifestyle changes	All participants: NB‐UVB phototherapy 3×/week for 12 weeks. All other treatment stopped.	12	12
Leite (2022)	Brazil	Psoriatic arthritis	32:33	*52.4 (12.8)*	54.5	‘Healthy diet’ with reduced saturated fat & increased MUFA/PUFA Placebo supplement If overweight or obese: hypocaloric diet (daily kcal intake = 500 kcal below BMR)	No lifestyle changes	No specific treatments, participants used a variety of psoriasis/psoriatic arthritis treatments	12	12
Lin (2022)	China	Psoriasis Type 2 diabetes	12:13	*55.9 (8.0)*	12.5	Once‐daily liraglutide injections (dose escalated depending on glycaemic control to maximum 1.8 mg/day)	Usual diabetes medications (excluding GLP‐1 agonists)	Control group only: oral acitretin and topical calcipotriol ointment	12	12
Naldi (2014)	Italy	Overweight or obese (BMI ≥ 25) Chronic plaque psoriasis (PASI ≥ 10)	151:152	53.0 (19.0)	29.0	2 phases: energy intake set at 0.8× RMR for 12 weeks, then 1.0× RMR for 8 weeks +Aerobic physical activity for 40 min ≥3×/week	15‐min informative session about the benefits of weight‐loss for improving psoriasis control	No specific treatments, participants used a mixture of systemic and topical treatments	20	20
Neema (2025)	India	Chronic plaque psoriasis (PASI ≥ 10)	60:60	*44.2 (13.8)*	33.5	Time‐restricted eating (‘intermittent fasting’, IF), 16‐h fast, 8‐h eating window	No lifestyle changes	All participants: methotrexate 0.3 mg/kg/week. Topical emollients also allowed	16	28^e^
Petković‐Dabić (2025)	Bosnia and Herze‐govina	Obese (BMI not specified) Chronic plaque psoriasis (PASI ≥ 10) Type 2 diabetes, diagnosed ≥6 months previously	15:16	*58.0 (10.7)*	19.4	Semaglutide 1.0 mg/kg/week Metformin at maximally‐tolerated dose (started prior to the study)	Metformin at maximally‐tolerated dose (started prior to the study)	Topical salicylic acid allowed in both groups	12	12

*Note*: Age (mean [SD]) shown in *italics* indicates values not reported for the overall group and derived from subgroup data using standard Cochrane formulas.

Abbreviations: BMI, body mass index; BMR, basal metabolic rate; BSA, body surface area; NAFLD, non‐alcoholic fatty liver disease; PASI, Psoriasis Area and Severity Index; REE, resting energy expenditure (also resting metabolic rate, RMR); TDR, total diet replacement (formula soups/shakes); blended TDR, TDR used alongside usual foods.

^a^ Number of participants enrolled at baseline.

^b^ Weight loss, PASI reduction and PASI 75 refer to the 24‐week (final) follow‐up; the intervention lasted 8 weeks.

^c^ Author‐provided data are at 24 weeks (end of intervention); no 52‐week data reported. The authors note that ~80% returned to baseline weight by the end of follow‐up.

^d^ A separate follow‐up publication 3 years later reported outcomes to 64 weeks in 32/60 participants;^42^ the table reports only the initial end of intervention (16‐week) outcomes.

^e^ In this study, methotrexate was stopped at 16 weeks for participants achieving PASI 50; those not achieving PASI 50 were withdrawn. To avoid confounding, only 16‐week data is included.

**TABLE 2:** Key findings of the included studies.

Study (Author, year)	Weight‐loss (%)^a^	PASI score change	DLQI change	PASI 50 achievement (%)	PASI 75 achievement (%)	PASI 100 achievement (%)	MDA achievement (%)	Itch score VAS change
Al‐Mutairi (2014)	13.1	Intervention: −27.5 (4.9) Control: −21.5 (5.3)	–	–	Intervention: 85.9^b^ Control: 59.4^b^	–	–	–
Faurschou (2015)	4.5	Intervention: −2.6 (2.1) Control: −1.3 (2.4)	Intervention: −2.5 (4.4) Control: −3.7 (4.8)	–	–	–	–	–
Gisondi (2008)	7.4	Intervention: −12.6 (6.3) Control: −6 (6.9)	–	Intervention: 86.7 Control: 48.3	Intervention: 66.7 Control: 29	–	–	–
Guida (2014)	11.4	Intervention: −5.1 (3.6) Control: −1.1 (3.8)	Intervention: −14.4 (1.9) Control: −2.2 (3.2)	–	–	–	–	Intervention: −13.6 (12.2) Control: −25.8 (30.4)
Ismail (2023)	6.3	Intervention: −2.1 (5.8) Control: −1.1 (8.5)	Intervention: −7.3 (5.9) Control: −1.8 (5.4)	–	–	–	–	–
Ismail (2024)	6.0	Intervention: −2.0 (4.85) Control: +0.2 (5.6)	–	–	–	–	–	–
Jensen (2013)	14.8	Intervention: −2.3 (0.7) Control: −0.3 (0.7)	Intervention: −2.7 (3.29) Control: −0.7 (3.3)	–	–	–	–	–
Kimball (2012)	8.0 (OD) 7.0 (SB)	Intervention (OD): −14.0 (5.8) Intervention (SB): −10.8 (5.8) Control: −9.4 (6.6)	–	–	Intervention (OD): 83 Intervention (SB): 56 Control: 38	–	–	–
Leite (2022)	0.3	Intervention: −0.8 (3.6) Control: −0.7 (3.0)	–	–	–	–	Intervention: 34.3 (+12% from baseline) Control: 27.3 (+9% from baseline)	–
Lin (2022)	7.3	Intervention: −12.3 (10.1) Control: −6.2 (3.4)	Intervention: −18.2 (5.9) Control: −8.5 (5.3)	Intervention: 90.9 Control: 9.1	Intervention: 72.7 Control: 27.3	–	–	–
Naldi (2014)	3.3	Intervention: −1.9 (1.9) Control: −1.0 (1.7)	–	Intervention: 49.7 Control: 34.2	Intervention: 24.5 Control: 19.1	Intervention: 16.6 Control: 10.5	–	–
Neema (2025)	1.4	Intervention: −9.38 (4.3) Control: −9.63 (6.81)	Intervention: −7.01 (3.91) Control: −7.12 (5)	Intervention: 82.5 Control: 78.6	–	–	–	–
Petković‐Dabić (2025)	12.4	Intervention: −10.75 (8.78) Control: −4.46 (9.03)	Intervention: −9 (5.87) Control: −2 (6.1)	–	–	Intervention: 8 Control: 0	–	–

*Note*: Values are mean (SD) unless stated otherwise.

Abbreviation: VAS, visual analogue scale.

^a^ Percentage weight loss for the intervention group.

^b^ PASI 75 achievement reported at final follow‐up (24 weeks), rather than at the end of the intervention (8 weeks), because data were available only for this time point.

Studies were conducted across nine countries (Table 1). Overall, 37.5% of participants were female. The mean (SD) age was 49.7 (12) years, and BMI was 30.8 (5.4) kg/m^2^.

### Interventions and comparators

Interventions lasted a mean (SD) of 14.4 (5.3) weeks, and the mean (SD) follow‐up was 15.5 (5.5) weeks. Four studies measured PASI beyond the intervention period.^40^, ^41^, ^42^, ^43^

Seven interventions involved dietary changes alone,^40^, ^43^, ^44^, ^45^, ^46^, ^47^ and four combined diet with physical activity.^41^, ^48^, ^49^, ^50^ Diets included low‐/very‐low‐energy, low‐fat, low‐carbohydrate, total dietary replacement and intermittent fasting. Two studies used liraglutide (≤1.8 mg/day), and one used semaglutide (1.0 mg/week).^51^, ^52^, ^53^ Most control participants were not advised to modify their diet or activity levels. However, three studies provided low‐intensity control interventions, comprising moderate physical activity 4×/week,^41^ group sessions to reinforce a normal, healthy diet^45^ or a one‐off informative session about the benefits of weight loss for improving psoriasis control.^48^

Four studies standardized psoriasis treatments for all participants,^41^, ^43^, ^46^, ^53^ while others allowed patients to use a variety of different treatments, regardless of their study group (Table 1). Lin et al. assigned different psoriasis treatments per study arm: the intervention group received liraglutide alone, while controls received pharmacotherapeutic usual care, comprising oral acitretin and topical calcipotriol.^46^, ^51^

### Risk of bias

**FIGURE 2:**
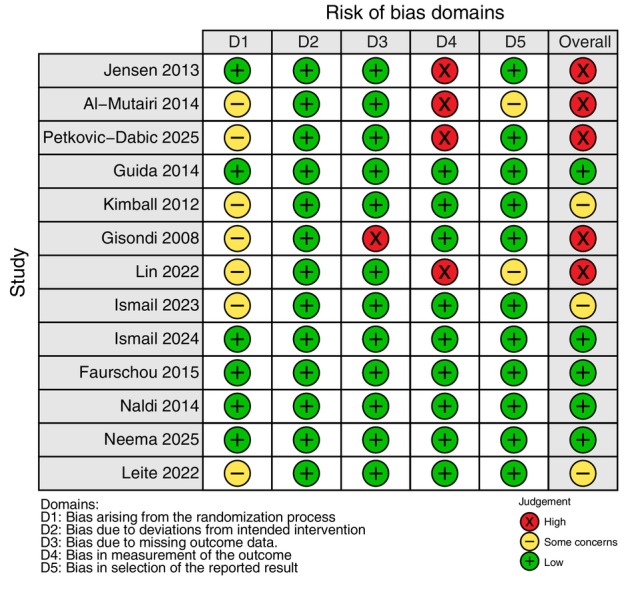
Risk of bias 2 (ROB2) assessment for included studies. Figure produced using robvis tool (https://www.riskofbias.info/welcome/robvis‐visualization‐tool).

Five studies were judged to have a high risk of bias,^40^, ^41^, ^45^, ^51^, ^53^ three judged to have some concerns^46^, ^47^, ^50^ and five low risk (Figure 2).^43^, ^44^, ^48^, ^49^, ^52^ The most common issue was inadequate reporting of randomization and/or allocation concealment in seven studies.^40^, ^41^, ^46^, ^47^, ^50^, ^51^, ^53^ There was no evidence of bias due to deviation from intended interventions (Table S3).

Some evidence of small‐study effects and possible publication bias, in the direction favouring the intervention, was observed upon examining funnel plots (Figure S1).

### GRADE certainty of evidence

The certainty of evidence was assessed to be high for PASI and DLQI, moderate for PASI50 and PASI75, and low for PASI100. ‘Serious’ concerns related to risk of bias and imprecision (Table S4).

### Process measure: weight change

All 13 studies (14 intervention arms and 1145 participants) were included in the weight change meta‐analysis. The pooled mean difference in weight change between the intervention and control groups was −6.9 kg (95%CI: −9.7 to −4.1, *I* ^2^ = 99.4%; Figure S2).

#### PASI

**FIGURE 3:**
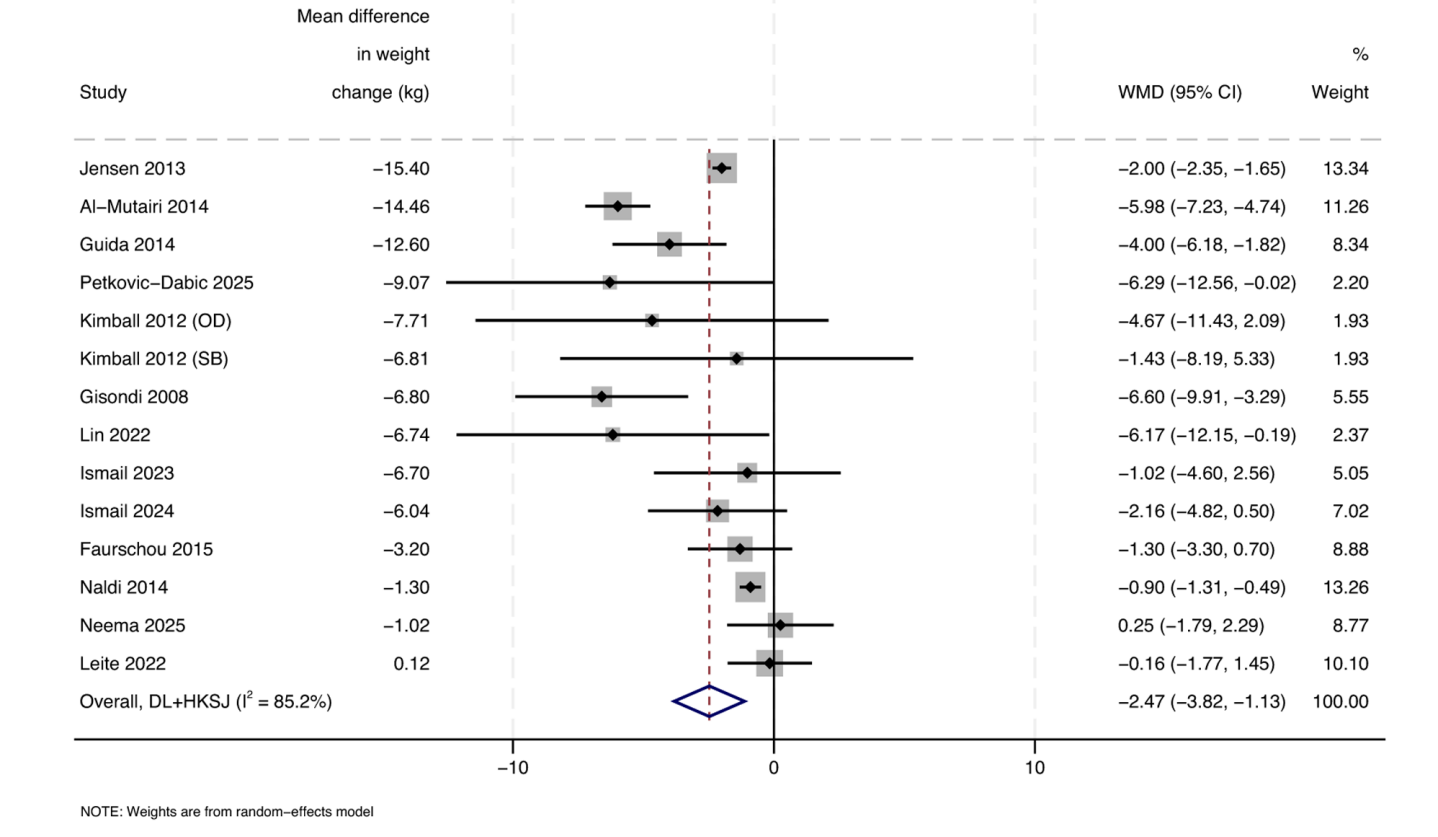
Forest plot comparing the change in PASI score between weight‐loss intervention and control groups, using a random effects model.

All 14 comparisons were included in our PASI analysis. Weight‐loss interventions were associated with a greater reduction in psoriasis severity than control, with a mean difference in PASI of −2.5 (95%CI: −3.8 to −1.1, *I* ^2^ = 85.2%; Figure 3). Excluding studies at high risk of bias decreased the effect size, with a pooled mean difference in PASI of −1.2 (95%CI: −2.2 to −0.2, *I* ^2^ = 32.9%; Figure S3). Excluding two studies that did not provide a measure of variance for the change in PASI did not meaningfully change the effect size, with a pooled mean difference in PASI of −1.8 (95%CI: −3.1 to −0.5, *I* ^2^ = 75.2%; Figure S4).^40^, ^46^

#### PASI50/75/100

Four comparisons, comprising 509 participants (253 intervention and 256 control), reported PASI50. Mean length of intervention/follow‐up was 18 weeks. Three comparisons used behavioural interventions, and one used liraglutide. Two comparisons were judged to be at high risk of bias. Mean difference in weight change between intervention and control groups was −4.0 kg. There was no statistically significant evidence of an effect of weight‐loss interventions on the likelihood of achieving PASI50 (RR = 1.5, 95% CI: 0.9–2.4, *I* ^2^ = 72.8%; Figure S6).

**FIGURE 4:**
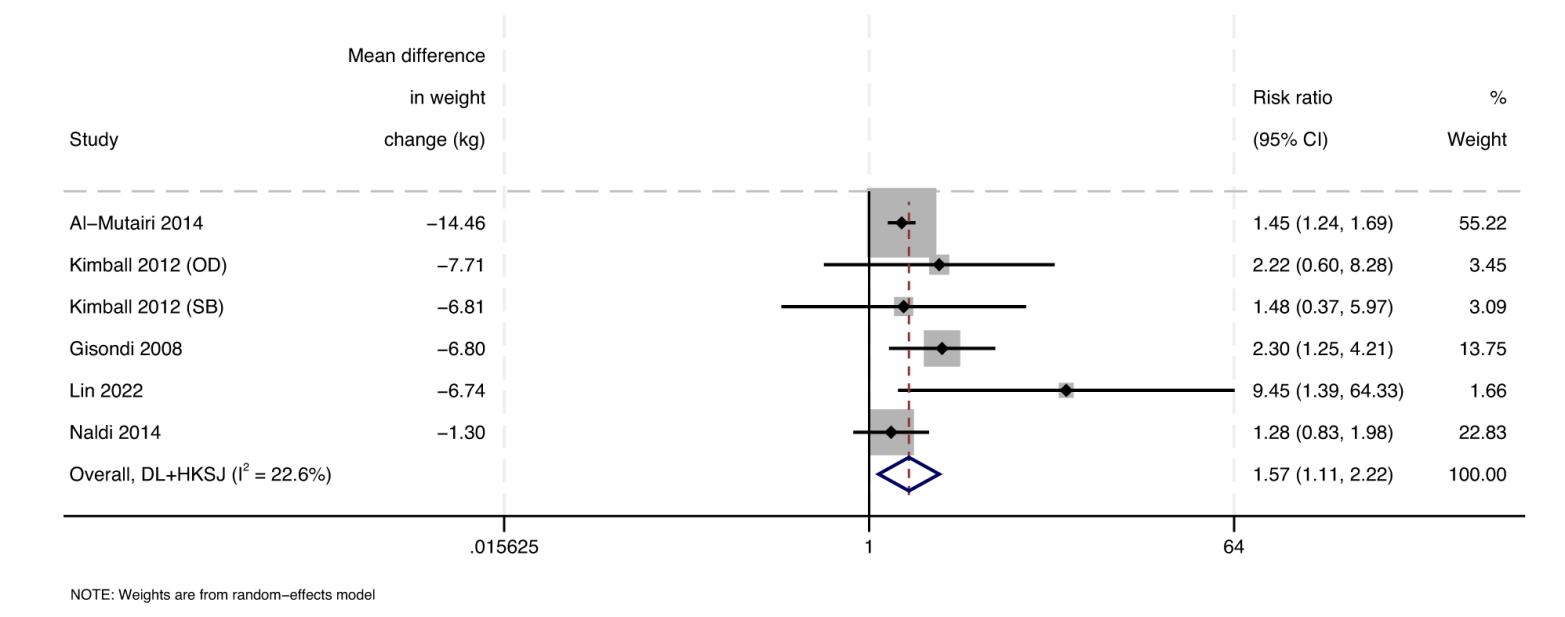
Forest plot demonstrating the risk of achieving PASI 75 in weight‐loss intervention participants compared to control participants.

Six comparisons, comprising 681 participants (344 intervention, 337 control), reported PASI75. Mean length of intervention and follow‐up was 15 weeks and 17 weeks, respectively. Four comparisons used behavioural interventions, and one used liraglutide. Mean difference in weight change between intervention and control groups was −7.3 kg. The analysis showed an improved likelihood of achieving PASI75 with weight‐loss interventions (RR = 1.6, 95%CI: 1.1–2.2, *I* ^2^ = 22.6%; Figure 4). Excluding three studies at high risk of bias had no meaningful change on the effect size, but the result was no longer statistically significant (Figure S5).

Two comparisons, comprising 334 participants (166 intervention and 168 control), reported PASI100. Mean length of intervention/follow‐up was 16 weeks. One comparison used a behavioural intervention, the other semaglutide. One comparison was judged to be at high risk of bias. Mean difference in weight change between intervention and control groups was −5.2 kg. There was no statistically significant evidence of increased likelihood of achieving PASI100 with weight‐loss interventions (RR = 1.62, 95%CI 0.27–9.73, *I* ^2^ = 0.0%; Figure S7).

#### DLQI

Seven comparisons, comprising 364 participants (182 intervention and 182 control), reported a change in DLQI. Mean length of intervention and follow‐up was 14 weeks. Four comparisons used behavioural interventions, and three used liraglutide or semaglutide. Mean difference in weight change between intervention and control groups was −7.8 kg. Three comparisons were judged to be at high risk of bias.

**FIGURE 5:**
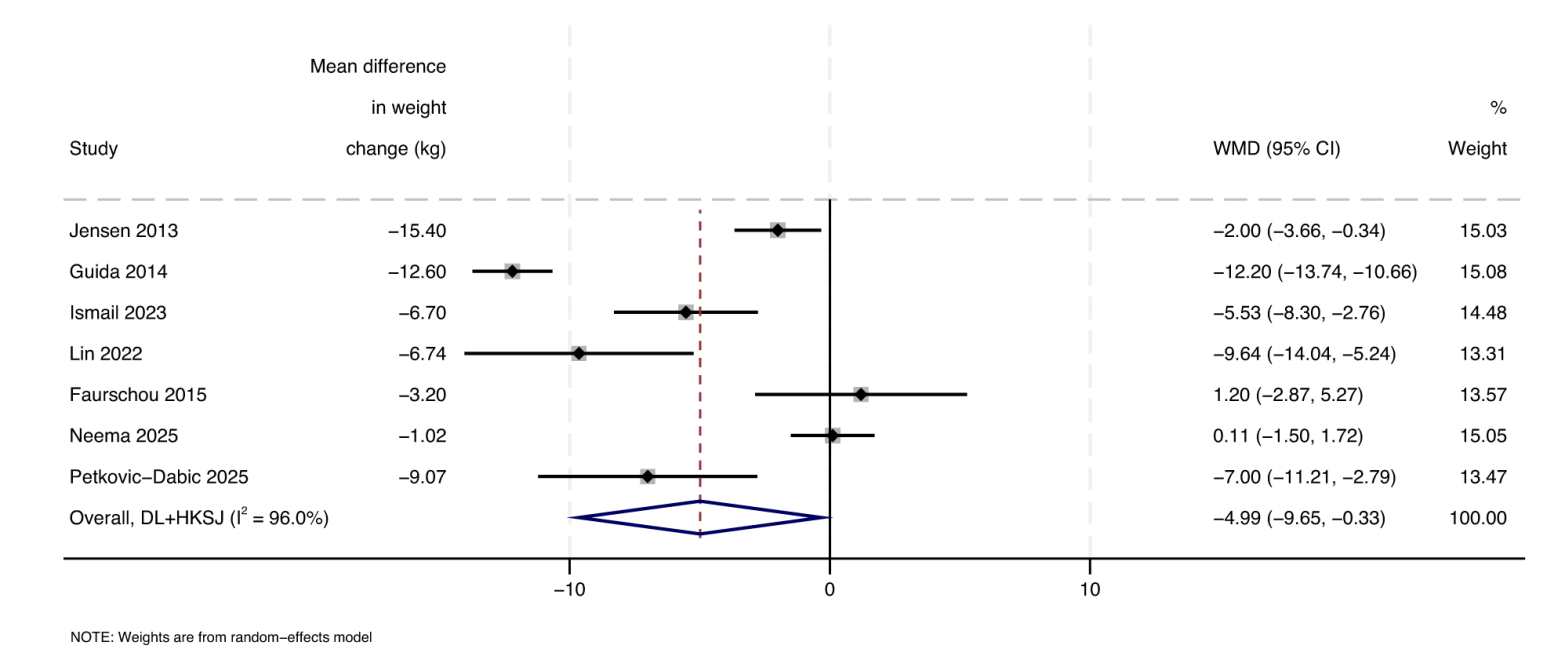
Forest plot to compare the change in DLQI score after a weight‐loss intervention compared to control, using a random effects model.

Weight‐loss interventions were associated with a greater improvement in DLQI compared to control, with a mean difference in DLQI of −4.99 (95%CI: −9.65 to −0.33, *I* ^2^ = 96.0%; Figure 5). Excluding studies at high risk of bias did not meaningfully change the effect size; however, the result was no longer statistically significant (Figure S8).

### Post‐hoc sensitivity analyses

Excluding studies with mild baseline psoriasis (PASI < 5) increased the pooled mean PASI difference to −3.1 (95%CI: −4.7 to −1.6, *I* ^2^ = 80.5%; Figure S9).^47^, ^48^ Excluding studies where the intervention achieved minimal weight loss compared to usual care (<1.5 kg difference between groups) also increased the pooled mean PASI reduction to −3.9 (95%CI: −5.4 to −2.3, *I* ^2^ = 81.6%; Figure S10).^43^, ^47^, ^48^, ^52^

Excluding one study, where psoriasis medical treatments were different between groups, did not meaningfully change the effect size upon PASI, PASI75, PASI50 or DLQI (Figures S11–S14); however, the DLQI result lost statistical significance.^51^

### Subgroup analyses

There was no evidence of between‐subgroup differences by intervention type for weight change (*p* = 0.475, Figure S15) or change in PASI (*p* = 0.829, Figure S16). There was also no evidence of a difference by intervention duration for change in PASI (*p* = 0.456, Figure S17).

In contrast, baseline PASI showed a significant between‐subgroup difference for change in PASI (*p* < 0.001), with larger improvements at higher baseline severity (Figure S18). Other outcomes had too few studies for subgroup analysis.

## DISCUSSION

### Key findings

Weight‐loss interventions reduce psoriasis severity (PASI), improve quality of life (DLQI), and increase the likelihood of achieving PASI75 for people with psoriasis and excess weight. Certainty of the evidence was high for PASI and DLQI, and moderate for PASI75.

### Strengths and limitations

We addressed an evidence gap by including RCTs testing any type of weight‐loss intervention in psoriasis. Collaboration with people with psoriasis helped to prioritize outcomes. This review is the first to meta‐analyse a quality‐of‐life measure regarding weight‐loss for patients living with psoriasis and assess evidence strength for each outcome.

The findings are limited by the small number of patients and studies for some outcomes, especially PASI50 and PASI100. We hypothesized that weight‐loss was the primary driver of psoriasis improvement, in addition to medical treatments, which were consistent across both groups in all except Lin et al.^51^ Therefore, we combined behavioural and pharmacological weight‐loss interventions and compared them with usual care/minimal interventions with the result that the pooled behavioural interventions varied in intensity, duration and delivery, contributing to weight‐loss variability and high heterogeneity. Differences in study populations (including baseline PASI, BMI and comorbidities) and control group advice also likely contributed to the observed heterogeneity. However, the findings from the primary analyses were not materially different in the sensitivity analyses. Furthermore, whilst comorbidities can affect psoriasis severity,^54^ these were consistent across study groups in studies which reported them (all except two^46^, ^48^), or listed as exclusion criteria. Several relevant studies which did not meet our inclusion criteria for this meta‐analysis also support our findings (Table S5).

Two planned analyses could not be conducted. First, a sensitivity analysis excluding trials where intervention participants received systemic psoriasis medications in addition to weight‐loss interventions was not feasible. Only one comparison permitted different psoriasis treatments between groups; the control group had access to topical calcipotriol and oral acitretin, while the intervention group received liraglutide alone.^51^ Excluding this study did not materially change the effect on any outcome. Second, a subgroup analysis stratifying comparator groups into ‘lower‐intensity intervention’ versus ‘usual care/minimal intervention’ was not performed, as all comparator groups fell into the latter category.

Several analyses were constrained by imprecision and inconsistency in the confidence intervals. The methods for addressing missing data were frequently inadequately reported, and the variability may have led to misleading differences between comparisons. Five comparisons were judged to be at high risk of bias, mostly due to concerns about assessor blinding, but removing these studies had no significant impact upon the magnitude of effect estimates. The quality of evidence was only judged to be high for PASI and DLQI. Additionally, no trials were conducted in primary or community care settings, where populations may differ from those recruited in specialist dermatology settings.

The long‐term sustainability of psoriasis improvement after weight loss is unclear due to generally short study follow‐ups. Two studies included in this review conducted extended follow‐up. One study demonstrated sustained benefits for up to 64 weeks,^42^ while the other reported weight regain and a relapse of psoriasis; however, this was concurrent with the discontinuation of ciclosporin therapy.^41^

### Clinical implications

The absolute PASI reduction was 2.5 (scale 0–72). Against a baseline mean (SD) PASI of 12.8 (7.6), this represents a proportionally meaningful improvement. The greatest benefits were seen in studies with higher baseline psoriasis (PASI > 5) and/or greater weight loss.^43^, ^47^, ^48^ Consistent with this, weight loss interventions were associated with an increased proportion of participants achieving PASI75. PASI50 and PASI100 were also more frequent in intervention groups, suggesting potential clinical benefit, but these analyses comprised fewer studies/participants and were not statistically significant.

In this review, the pooled mean (SD) weight loss in intervention groups was −6.5 kg (7.4). Advice without referral or support generally leads to only around 1 kg weight loss at 1 year.^55^, ^56^ Weight loss achieved in supported programmes varies by intervention type. Behavioural approaches incorporating modest energy deficits, with or without exercise, typically lead to weight loss of 4 kg in 1 year.^57^ Total dietary replacement programmes are more effective, usually achieving 10 kg weight loss after 1 year.^58^, ^59^ Based on the results of this review, programmes such as these will likely confer clinical benefit to people with psoriasis.

Two interventions in this review used liraglutide (≤1.8 mg/day), below the 3 mg/day dose recommended for weight loss, and neither reported structured behavioural support.^51^, ^52^, ^60^, ^61^, ^62^ These features likely contributed to the modest weight loss compared with that observed in obesity treatment trials.^63^ Newer GLP‐1 agents achieve greater losses; in the trial using semaglutide, the dose was escalated to 1 mg/week within a 12‐week programme and produced substantially larger weight loss.^53^, ^64^, ^65^ No RCTs evaluated bariatric surgery, which typically yields greater and more durable weight reduction. Observational cohorts report a 59% mean PASI improvement (95%CI: 42–74%) after bariatric surgery, with the magnitude of weight loss predicting clinical response.^66^, ^67^

The pooled mean difference in DLQI favoured weight‐loss interventions by almost 5 points (scale 0–30). Given widely used DLQI Minimally Clinically Important Difference (MCID) estimates of ~3–5 points, this magnitude is clinically significant.^3^, ^68^ This may reassure clinicians hesitant to recommend weight‐loss interventions.^20^, ^21^, ^22^

### Discussion with people living with psoriasis

Our patient advisory group reported that these findings would motivate them to consider weight loss as an adjunct psoriasis treatment. Many said the results mirrored their experience that psoriasis severity fluctuates with body weight. They valued the combined improvements in clinical outcomes and quality of life, which they feel provide meaningful motivation and encourage engagement in weight‐loss programmes. Patients expressed frustration that discussions on diet and weight management were infrequent in psoriasis‐related medical consultations.

## CONCLUSIONS

Weight‐loss interventions may improve skin disease and quality of life for people with psoriasis and excess weight. Clinicians should consider using these findings to counsel patients and refer them for weight‐loss support when appropriate. Future research should bridge the gap between evidence and patient awareness, while addressing clinician hesitancy. Understanding how to implement these findings in real‐world settings, such as dermatology and primary care, is essential.

## AUTHOR CONTRIBUTIONS

**Sarah Morrow:** conceptualizsation, data curation, formal analysis, funding acquisition, methodology, visualization, writing – original draft preparation, writing – review and editing. **Jadine Scragg:** conceptualization, data curation, formal analysis, funding acquisition, methodology, supervision, writing – review and editing. **Poppy Hawkins:** data curation, writing – review and editing. **Christopher E. M. Griffiths:** conceptualization, funding acquisition, supervision, writing – review and editing. **Thanasis G. Tektonidis:** conceptualization, supervision, writing – review and editing. **Eli Harriss:** conceptualizsation; data curation; **Susan Jebb:** conceptualization, funding acquisition, supervision, writing – review and editing.

## FUNDING INFORMATION

This research was jointly funded by the British Skin Foundation (003_BSFBAD_23) and the National Institute for Health and Care Research (NIHR304207). JS and SAJ are supported by the NIHR Oxford Biomedical Research Centre (NIHR203311). The funders had no role in study design, data collection, data analysis, manuscript preparation or decision to publish.

## CONFLICT OF INTEREST STATEMENT

Dr Sarah Morrow: Research grants received from the British Skin Foundation and the NIHR. Dr Poppy Hawkins: Received PhD studentship funding from CAFEM, University of Hertfordshire, and personal consulting fees from UCB, outside the submitted work. Prof Christopher E.M. Griffiths: Received institutional funding from Almirall pharmaceutical company. Received personal consulting fees from Boehringer Ingelheim, Boots UK, Bristol Myers Squibb, Evelo Bioscience, Inmagene, Johnson & Johnson, Novartis and Nxera. Received personal payment for speaking at educational events from AbbVie, Almirall, Boehringer Ingelheim, Bristol Myers Squibb, Johnson & Johnson, Lilly, Novartis and UCB. Received support for attending meetings from Amryt Pharma, Novartis and Johnson & Johnson. Participated on the board for Artax. Director of the Global Psoriasis Atlas. Founding stock of The Skin Diary. All outside the submitted work. Dr Thanasis G Tektonidis: None. Eli Harriss: None. Dr Jadine Scragg: Salary part funded by the NIHR Biomedical Research Centre and the NovoNordisk Foundation outside the submitted work. Prof Susan Jebb: Received institutional research grant from the NIHR Biomedical Research Centre, outside the submitted work. Provision of weight management intervention to the NHS for investigator‐led research from Oviva and provision of weight management intervention for an investigator‐led research study from Second Nature, both outside the submitted work.

## ETHICAL APPROVAL

Not applicable. This systematic review used only published data and did not involve human participants or animals; ethics approval and consent were not required.

## ETHICS STATEMENT

Not applicable.

## Supporting information


Data S1.

Data S2.

Appendix S1.

## Data Availability

Data are from published research and therefore in the public domain. Aggregate data may be available on request to Dr Morrow, sarah.morrow@phc.ox.ac.uk.
