# Supplementary material for: Impact of weight‐loss interventions on psoriasis severity: A systematic review and meta‐analysis
Source: J Eur Acad Dermatol Venereol. 2025 Dec 19;40(6):980–93. doi: 10.1111/jdv.70247 (PMC13206337; doi:10.1111/jdv.70247)
Supplement: Supplementary file 1 — Data S1. [file JDV-40-980-s003.docx]

**Table S1:** Details regarding data extraction and handling

| **Category** | **Methods / comments** | **Studies affected** |
| --- | --- | --- |
| General assumptions & transformations | Inverse‑variance meta‑analysis of continuous outcomes.  Where needed, transformed summary statistics to obtain means/SDs on a common scale per Cochrane guidance.  When studies reported both change‑from‑baseline and post‑intervention values, we analysed a single form per meta‑analysis (i.e., did not mix within an analysis). | All included studies (general rule) |
| Imputed change and calculated correlation coefficients^1^ | Where baseline and final means/SDs were reported but mean difference and SD of change were not, mean change was computed (final − baseline).  SD of change was imputed using the within‑participant correlation ρ:  SD_change = sqrt(SD_baseline^2 + SD_final^2 − 2·ρ·SD_baseline·SD_final).  (ρ was derived from reported data) | Studies reporting baseline & final only (no change SD) |
| Missing SDs: from standard errors^2^ | Where SEs were reported, SD was calculated as SD = SE × √n. | Jensen *et al.* |
| Missing SDs: from interquartile range (IQR)^3^ | Where only IQRs were reported, SDs were approximated assuming normality as SD ≈ IQR/1.35 (per Cochrane). | Naldi *et al.;* Jensen *et al.* |
| Missing variability^4^ | For change in PASI reported with no measure of variance, SDs were imputed using the mean of SDs from other eligible studies reporting the same outcome/type of intervention/time‑point *(Cochrane: borrowing SDs when necessary^4^; Furukawa et al. 2006**).* This preserved study inclusion given the small evidence base.  To test the effect of including these studies, a sensitivity analysis was performed to exclude the relevant studies (Figure S4). | Al-Mutairi *et al.*  Kimball *et al.* |
| Multi‑arm trial handling (shared control)^5^ | To avoid double‑counting a shared control where two intervention arms were compared with one control, the control group was split evenly between comparisons (n halved; means/SDs unchanged), in line with Cochrane advice for multiple‑arm studies. | Kimball *et al.* |
| Imputation: mean from median^6^ | Where only median was reported and n≥25, the mean was approximated as the median *(Hozo et al. 2005)**  Where only median was reported and n<25, if there was no range (minimum and maximum values) provided, the mean was also approximated as the median, assuming plausible symmetry. | Jensen *et al.*  Naldi *et al.*  Petkovic-Dabic *et al.* |
| Unit conversions | Body weights reported in pounds were converted to kilograms using kg = lb / 2.205. | Kimball *et al.* |
| BMI converted to weight (kg) | Three studies provided final timepoint BMI but not body weight. In these cases, the average height of the relevant population was used to calculate body weight from BMI, using the equation: weight(kg)=BMI*height(m)^2^ | Petkovic-Dabic *et al.*  Ismail *et al.* 2023  Ismail *et al.* 2024 |
| Weight (kg) converted to BMI | In two studies, body weight at baseline and follow-up was provided, but not BMI. Whilst the BMI data was not meta-analysed, it was used to describe the participant populations for the various meta-analyses. Therefore in these cases, BMI was calculated from weight using the equation: BMI=weight(kg)/height(m)^2^ | Kimball *et al.*  Neema *et al.* |

*Table demonstrates the rules applied to extract/transform outcomes (weight, PASI, DLQI), derive missing statistics, and address multi‑arm designs. Methods follow Cochrane Handbook guidance; study‑specific exceptions are noted.*

*Key formulas and procedures are from the Cochrane Handbook (current online version, last checked 02/09/2025), plus additional relevant papers:*

*^1^Section 6.5.2.8 (imputing SD of change using within‑participant correlation ρ)*

*^2^Chapter 6, Section 6.5.2.2 (deriving SD from SE/CI)*

*^3^Section 6.5.2.5 (IQR ≈ 1.35×SD)*

*^4^Section 6.5.2.7 (when SDs remain missing, imputation options)*

*^5^Multi‑arm studies and shared groups are covered in Chapter 23 (Including variants on randomised trials)*

*^6^Section 6.5.2.9 (missing means)*

**Approximating means from medians (mean≈median for n≥25) follows Hozo SP, Djulbegovic B, Hozo I. BMC Med Res Methodol. 2005;5:13.*

***Imputation of SDs by borrowing from other studies is supported by Furukawa TA et al. J Clin Epidemiol. 2006;59:7–10.*

**Table S2:** Handling of missing data in each study

| **Study**  **(Author, year)** | **Total n randomised** | **N analysed** | **% lost to follow-up** | **Missing data handling** |
| --- | --- | --- | --- | --- |
| Al-Mutairi 2014 | 262 | 262 | 0 | No missing data |
| Faurschou 2015 | 20 | 20 | 0 | No missing data |
| Gisondi 2008 | 61 | 43 | 30 | ITT analysis |
| Guida 2014 | 44 | 36 | 18 | ITT analysis |
| Ismail 2023 | 64 | 60 | 6 | Complete case analysis |
| Ismail 2024 | 60 | 60 | 0 | No missing data |
| Jensen 2013 | 60 | 53 | 12 | BOCF |
| Kimball 2012 | 30 | 20 | 33 | ITT analysis |
| Leite 2022^a^ | 65 | 62 | 5 | ITT analysis |
| Lin 2022 | 25 | 24 | 8 | Complete case analysis |
| Naldi 2014 | 303 | 282 | 7 | ITT analysis |
| Neema 2025 | 120 | 113 | 6 | Complete case analysis |
| Petkovic-Dabic 2025 | 31 | 28 | 10 | Complete case analysis |

*^a^ Numbers exclude participants in trial arms which were not included in the current review*

*ITT = Intention to treat analysis. BOCF = Baseline observation carried forward.*

**Table S3:** Risk of Bias 2 (RoB2) – Reasons for non-low‑risk judgements by domain

| **Domain (RoB 2)** | **Studies judged High risk / Some concerns** | **Reason(s) for judgement** |
| --- | --- | --- |
| Domain 1: Bias arising from the randomisation process | Al‑Mutairi et al., Petkovi$\acute{c}$-Dabi$\acute{c}$ et al., Kimball et al., Gisondi et al., Ismail et al. (2023), Lin et al., Leite et al. (Some concerns) | In all seven studies, there was insufficient information on allocation concealment. Concerns did not generally relate to the random sequence generation itself but to the lack of detail on how / whether allocation was concealed. |
| Domain 2: Bias due to deviations from intended interventions | Not applicable | All studies were judged to be ‘low-risk’ for this domain. Whilst participants in many studies were aware of their study group, in no case did this appear to have led to deviations from intended intervention. |
| Domain 3: Bias due to missing outcome data | Gisondi et al. (High risk) | Markedly unequal withdrawal rates between groups (intervention 4/30, 13.3% vs control 14/31, 45.1%), likely to have affected the overall result; hence ‘high risk’. |
| Domain 4: Bias in measurement of the outcome | Jensen et al., Al‑Mutairi et al., Petkovi$\acute{c}$-Dabi$\acute{c}$ et al., Lin et al. (High risk) | Outcome assessors were, or were likely to have been, aware of group allocation. Two studies did not report assessor blinding (Al‑Mutairi et al.; Lin et al.), and two explicitly stated that PASI was assessed by unblinded investigators (Jensen et al.; Petkovi$\acute{c}$-Dabi$\acute{c}$ et al.). Given that PASI requires assessor judgement, knowledge of allocation could feasibly influence ratings. |
| Domain 5: Bias in selection of the reported result | Al‑Mutairi et al. and Lin et al. (Some concerns) | No information on analysis according to a pre‑specified statistical analysis plan and no trial registry entry referenced. All other studies referenced a registry entry and reported pre‑specified outcomes (therefore ‘low risk’ for those studies). |

Only studies judged ‘High risk’ or ‘Some concerns’ are listed for each domain; all others were ‘Low risk’.

‘Some concerns’ denotes potential for bias; ‘High risk’ indicates bias likely to affect results. Judgements follow the RoB 2 framework.

PASI = Psoriasis Area and Severity Index.

**Table S4:** GRADE assessment

| **Outcome** | **Effect estimate**  ***(95% CI)*** | **No. of participants**  ***(no. of comparisons)*** | **Certainty of the evidence (GRADE)** | | | | | **Certainty (Quality) of the evidence** |
| --- | --- | --- | --- | --- | --- | --- | --- | --- |
|  |  |  | **Risk of bias** | **Inconsistency** | **Indirectness** | **Imprecision** | **Publication bias** |  |
| PASI | MD: -2.47  *(-3.8 to -1.1)* | 1145  *(14)* | Not Serious | Not Serious | Undetected | Not Serious | Not serious | High^1^ |
| PASI 50 | RR 1.5  *(0.9 to 2.4)* | 509  *(4)* | Not Serious | Not serious | Undetected | Serious | Not Serious | Moderate^2^ |
| PASI 75 | RR 1.57  *(1.11 to 2.22)* | 681  *(6)* | Serious | Not Serious | Undetected | Not Serious | Not Serious | Moderate^3^ |
| PASI 100 | RR 1.62  (0.27 to 9.73) | 334  *(2)* | Not serious | Undetected | Undetected | Serious | Not serious | Low^4^ |
| DLQI | MD: -4.99  *(-9.65 to -0.33)* | 364  *(7)* | Not Serious | Not Serious | Undetected | Not serious | Not Serious | High^5^ |

***GRADE Working Group grade of evidence***

*High certainty: we are very confident that the true effect lies close to that of the estimate of the effect.*

*Moderate certainty: we are moderately confident in the effect estimate: The true effect is likely to be close to the estimate of the effect, but there is a possibility that it is substantially different.*

*Low certainty: our confidence in the effect estimate is limited: the true effect may be substantially different from the estimate of the effect.*

*Very low certainty: we have very little confidence in the effect estimate: the true effect is likely to be substantially different from the estimate of effect.*

*^1^* ***PASI:*** *Risk of bias: Downgraded by 1 level. 5/13 studies were at high risk of bias, excluding these studies did not meaningfully affect the result; Imprecision: Substantial heterogeneity (I^2^=85%) but many reasons for this (different interventions and populations), generally overlapping confidence intervals and point estimates show similar trend; Imprecision: A few studies’ confidence intervals cross the line of no effect, but the confidence interval for the overall analysis does not. Overall confidence interval is small. High number of participants overall. Publication bias: Downgraded by 1 level because of some mild asymmetry on funnel plots, however no evidence from trial registries that trials have not been published.*

*^2^* ***PASI 50:*** *Risk of bias: Downgraded by 2 levels as 2/4 studies at high risk of bias, but largest study (Naldi et al.) is low-risk; Inconsistency: Downgraded by 1 level due to I^2^=73%, however confidence intervals overlapping and point estimates similar. Imprecision: Downgraded by 2 levels because the overall confidence interval crosses the line of no effect. Publication bias: Downgraded by 1 level because there were insufficient studies to assess for publication bias, however no evidence from trial registries that trials have not been published.*

*^3^* ***PASI 75:*** *Risk of bias: Downgraded by 2 levels as 3/5 studies at high risk of bias, and one of these is particularly large; Inconsistency: Downgraded by 1 level because one point estimate is meaningfully different from the others, however confidence intervals all overlap; Imprecision: Some studies’ confidence intervals cross the line of no effect, but the confidence interval for the overall effect does not; Publication bias: Downgraded by 1 level because there were insufficient studies to assess for publication bias, however no evidence from trial registries that trials have not been published.*

*^4^* ***PASI 100:*** *Risk of bias: Downgraded by 1 level because one of the 2 studies is at high risk of bias, but this is a much smaller study than the one at low-risk. Imprecision: overall confidence interval crosses the line of no effect. Publication bias: Downgraded by 1 level because there were insufficient studies to assess for publication bias, however no evidence from trial registries that trials have not been published.*

*^5^* ***DLQI:*** *Risk of bias: Downgraded by 1 level because 3/7 studies at high risk of bias but all relatively small sizes; Inconsistency: Downgraded by 1 level because of high heterogeneity, however clear reasons for this (different interventions, different populations, different durations). Generally overlapping confidence intervals. Imprecision: Overall confidence interval does not cross the line of no effect, although 2 studies’ confidence intervals do. Wide overall CI due to heterogeneity. Publication bias: Downgraded by 1 level because there were insufficient studies to assess for publication bias, however no evidence from trial registries that trials have not been published.*

**Table S5:** Studies which almost met inclusion criteria

| Author | Study Focus | Reason for Exclusion | Key Findings |
| --- | --- | --- | --- |
| Di Minno et al.^1^ | Effect of weight-loss on achieving minimal disease activity in psoriatic arthritis patients starting TNFα-blockers | Did not provide final data regarding skin severity | Greater weight-loss associated with higher rates of minimal disease activity: 23.1% for <5% weight-loss, 44.8% for 5-10%, and 59.5% for >10% |
| Thomsen et al.^2,3^ | High-intensity interval training for psoriatic arthritis | The intervention was not specifically aiming to achieve weight loss. Aim of intervention was to improve cardiovascular fitness. | Improvements in cardiovascular fitness, reduced truncal fat mass and reduced fatigue, but no significant difference in final PGA between groups |
| Del Giglio et al.^4^ | Effect of weight-loss to maintain remission in psoriasis patients with obesity following systemic psoriasis treatment cessation | Aimed to maintain remission rather than to treat psoriasis | No statistically significant difference in psoriasis remission maintenance, although the trend favoured the intervention group. |
| Landgren et al.^5^ | Explored effect of a very-low-energy diet in psoriatic arthritis patients | Control group did not have psoriasis | Significant positive correlation between weight-loss and reduced disease activity |
| Lambadiari et al.^6^ | Very-low-calorie ketogenic diet vs. hypocaloric Mediterranean diet for psoriatic arthritis (crossover trial) | Intensity of the interventions was the same (same energy deficit in both) | Ketogenic diet led to significant improvements in PASI and Disease Activity in PSoriatic Arthritis score (DAPSA). Mediterranean diet did not. |

**Supplementary references**

1 Di Minno MN, Peluso R, Iervolino S *et al.* Weight loss and achievement of minimal disease activity in patients with psoriatic arthritis starting treatment with tumour necrosis factor α blockers. *Ann Rheum Dis* 2014; **73**: 1157-62.

2 Thomsen RS, Nilsen TIL, Haugeberg G *et al.* Impact of High-Intensity Interval Training on Disease Activity and Disease in Patients With Psoriatic Arthritis: A Randomized Controlled Trial. *Arthritis Care Res (Hoboken)* 2019; **71**: 530-7.

3 Thomsen RS, Nilsen TIL, Haugeberg G *et al.* Effect of high-intensity interval training on cardiovascular disease risk factors and body composition in psoriatic arthritis: a randomised controlled trial. *RMD Open* 2018; **4**: e000729.

4 Del Giglio M, Gisondi P, Tessari G *et al.* Weight reduction alone may not be sufficient to maintain disease remission in obese patients with psoriasis: a randomized, investigator-blinded study. *Dermatology* 2012; **224**: 31-7.

5 Landgren AJ, Jonsson CA, Bilberg A *et al.* Serum IL-23 significantly decreased in obese patients with psoriatic arthritis six months after a structured weight loss intervention. *Arthritis Res Ther* 2023; **25**: 131.

6 Lambadiari V, Katsimbri P, Kountouri A *et al.* The Effect of a Ketogenic Diet versus Mediterranean Diet on Clinical and Biochemical Markers of Inflammation in Patients with Obesity and Psoriatic Arthritis: A Randomized Crossover Trial. *Int J Mol Sci* 2024; **25**.
