# Supplementary material for: Impact of weight‐loss interventions on psoriasis severity: A systematic review and meta‐analysis
Source: J Eur Acad Dermatol Venereol. 2025 Dec 19;40(6):980–93. doi: 10.1111/jdv.70247 (PMC13206337; doi:10.1111/jdv.70247)

# Supplementary Figures

**FIGURE S1:** Funnel plot for included studies, based upon the change in PASI scores in the different studies, assuming fixed effects model (**A**) and random-effects model (**B**).


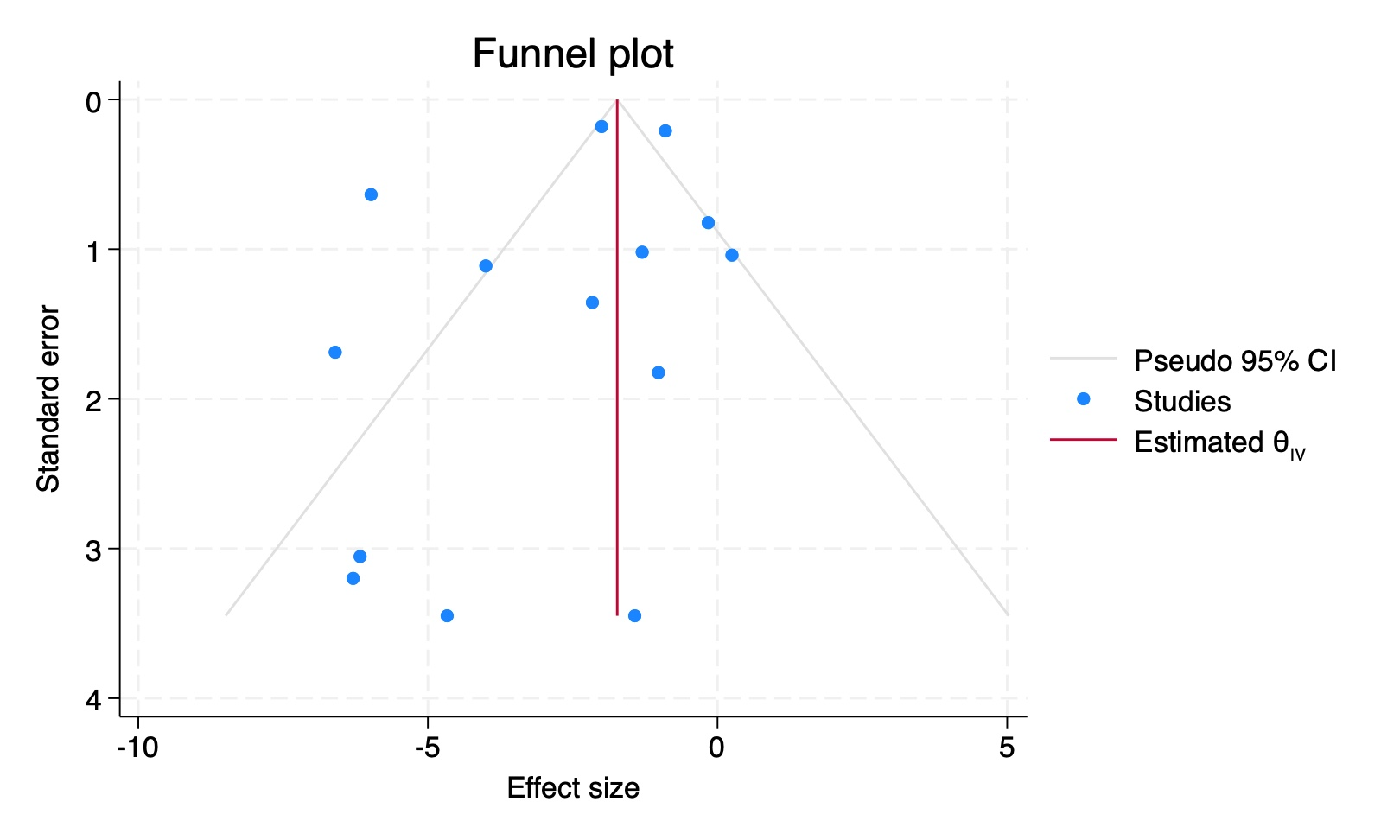


A


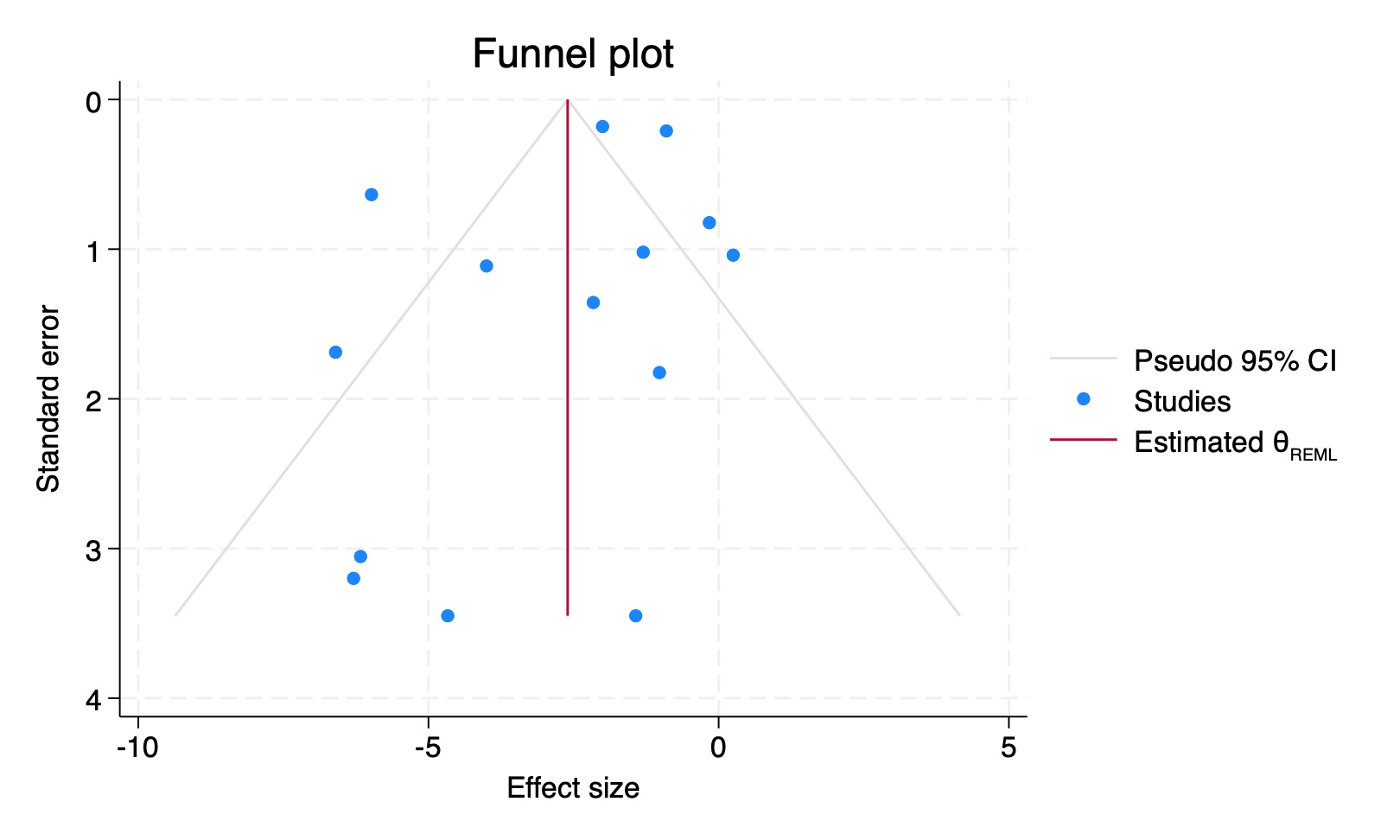


B

**FIGURE S2:** Forest plot to show the mean difference in weight change (kg) between intervention groups vs control groups, using the random effects model.


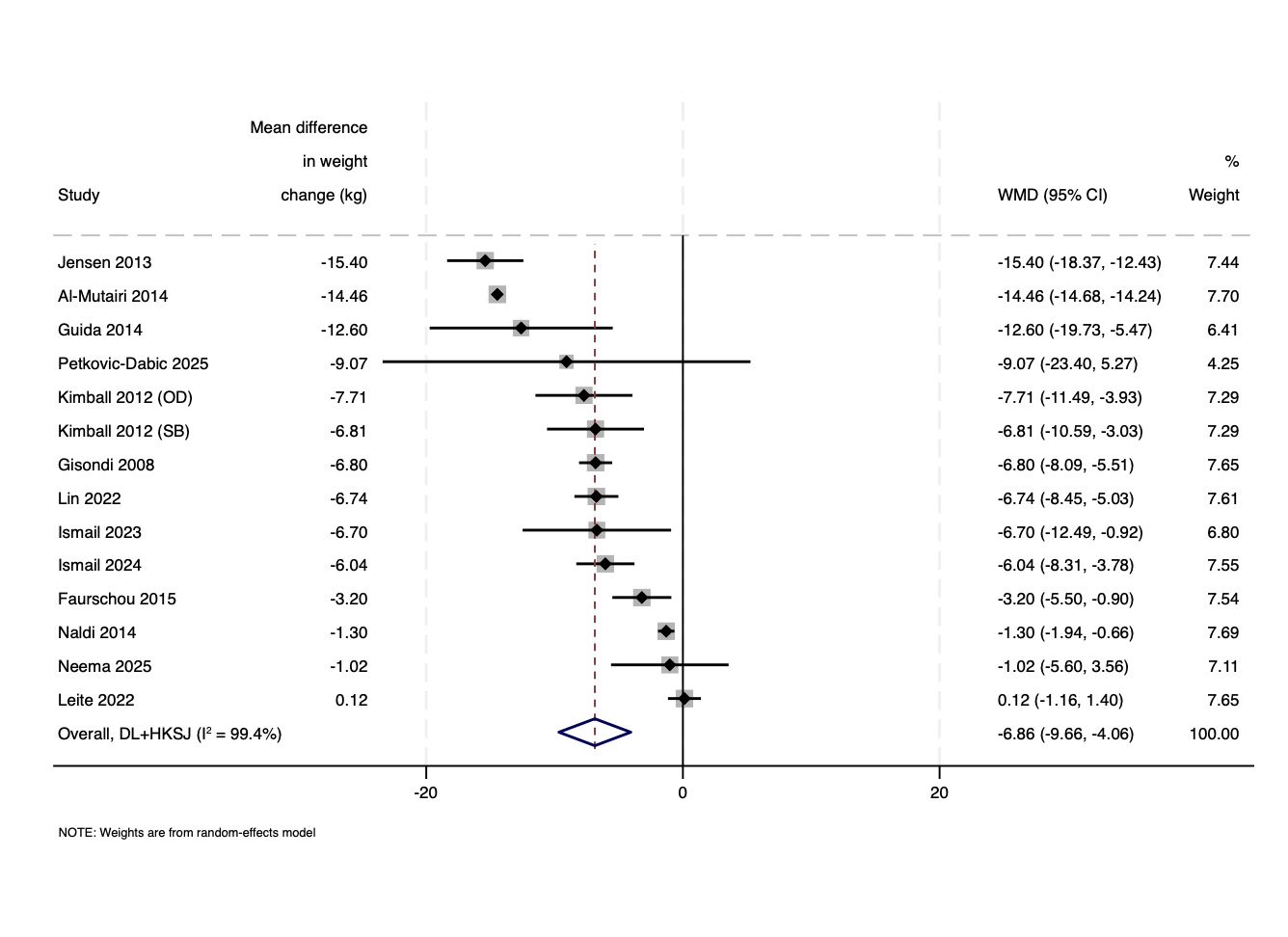


**FIGURE S3:** Sensitivity analysis. Forest plot to compare the change in PASI score after a weight-loss intervention compared to control, using the random effects model, excluding studies which were judged to be at high risk of bias.


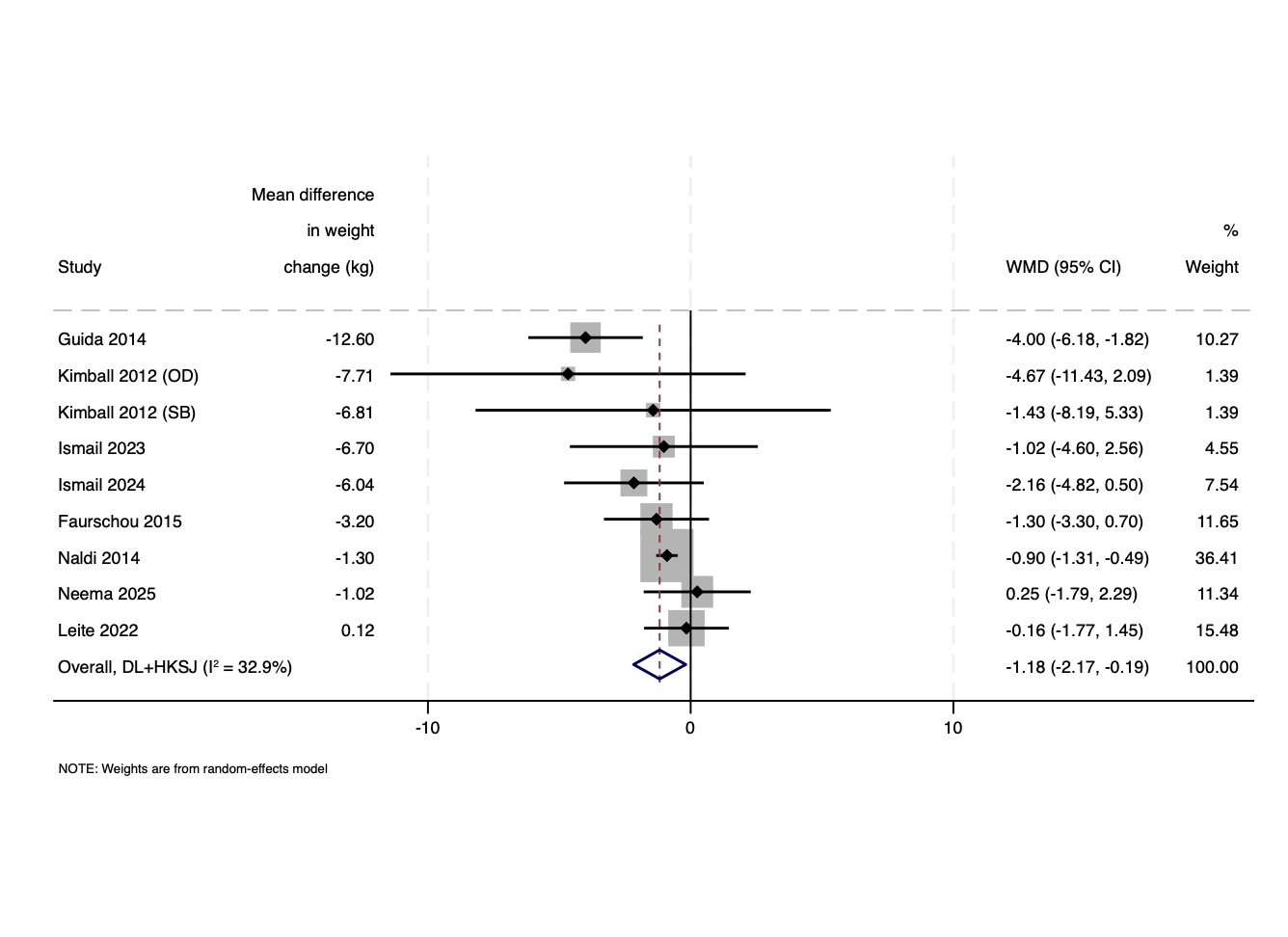


**FIGURE S4:** Sensitivity analysis. Forest plot to compare the change in PASI after a weight-loss intervention compared to control, using the random effects model, excluding two studies where a measure of variance was not provided for the change in PASI, and this therefore had to be imputed.


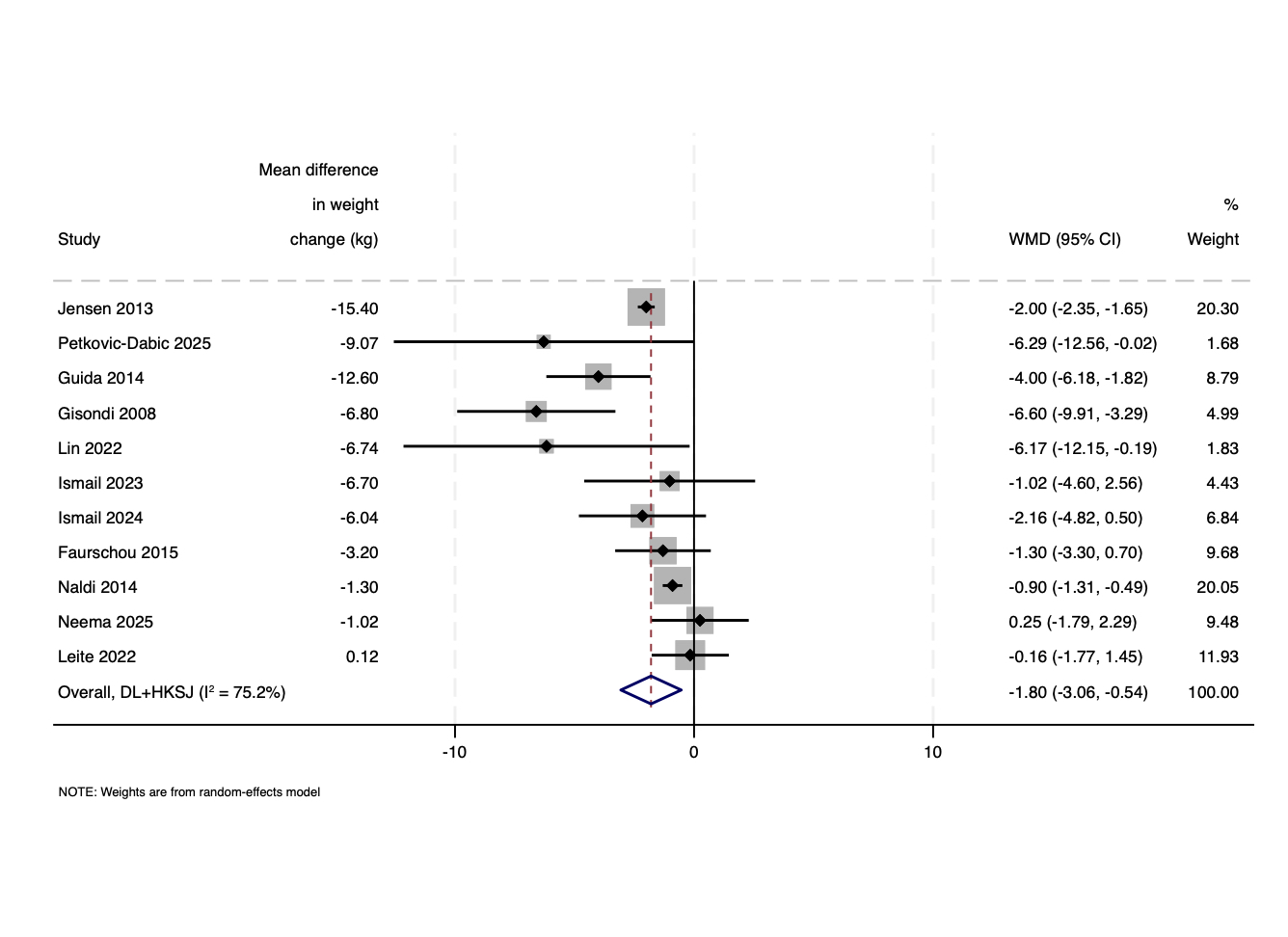


**FIGURE S5:** Sensitivity analysis. Forest plot demonstrating the risk of achieving PASI 75 in weight-loss intervention participants compared to control participants, excluding studies at high risk of bias.


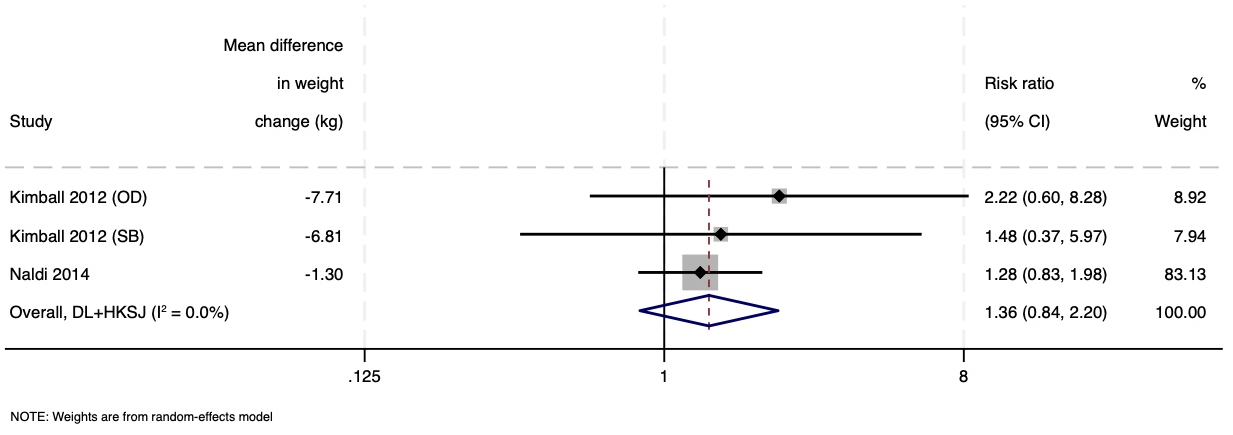


**FIGURE S6:** Forest plot demonstrating the risk of achieving PASI 50 in weight-loss intervention participants compared to control participants.


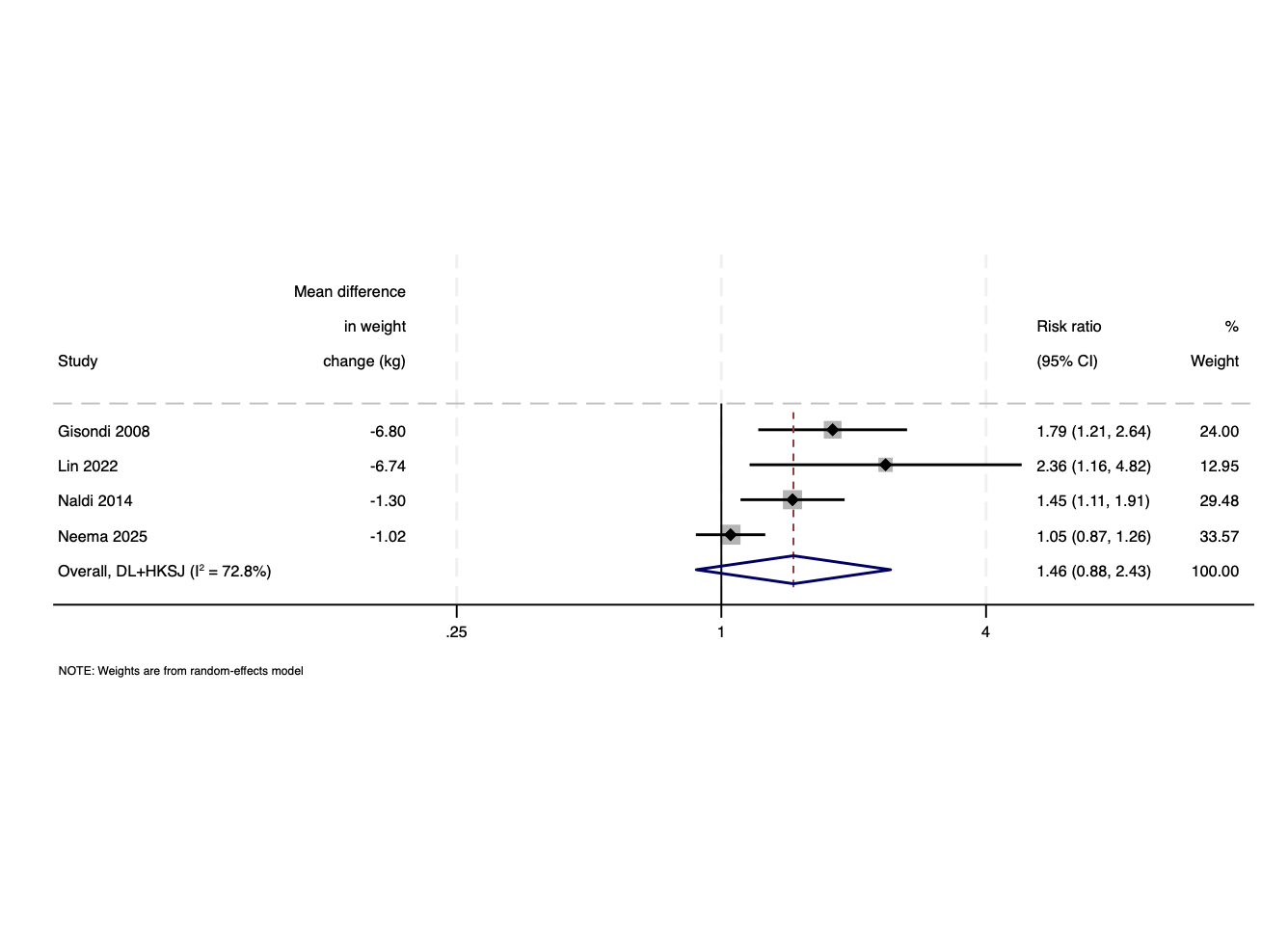


**FIGURE S7:** Forest plot demonstrating the risk of achieving PASI 100 in weight-loss intervention participants compared to control participants.

**
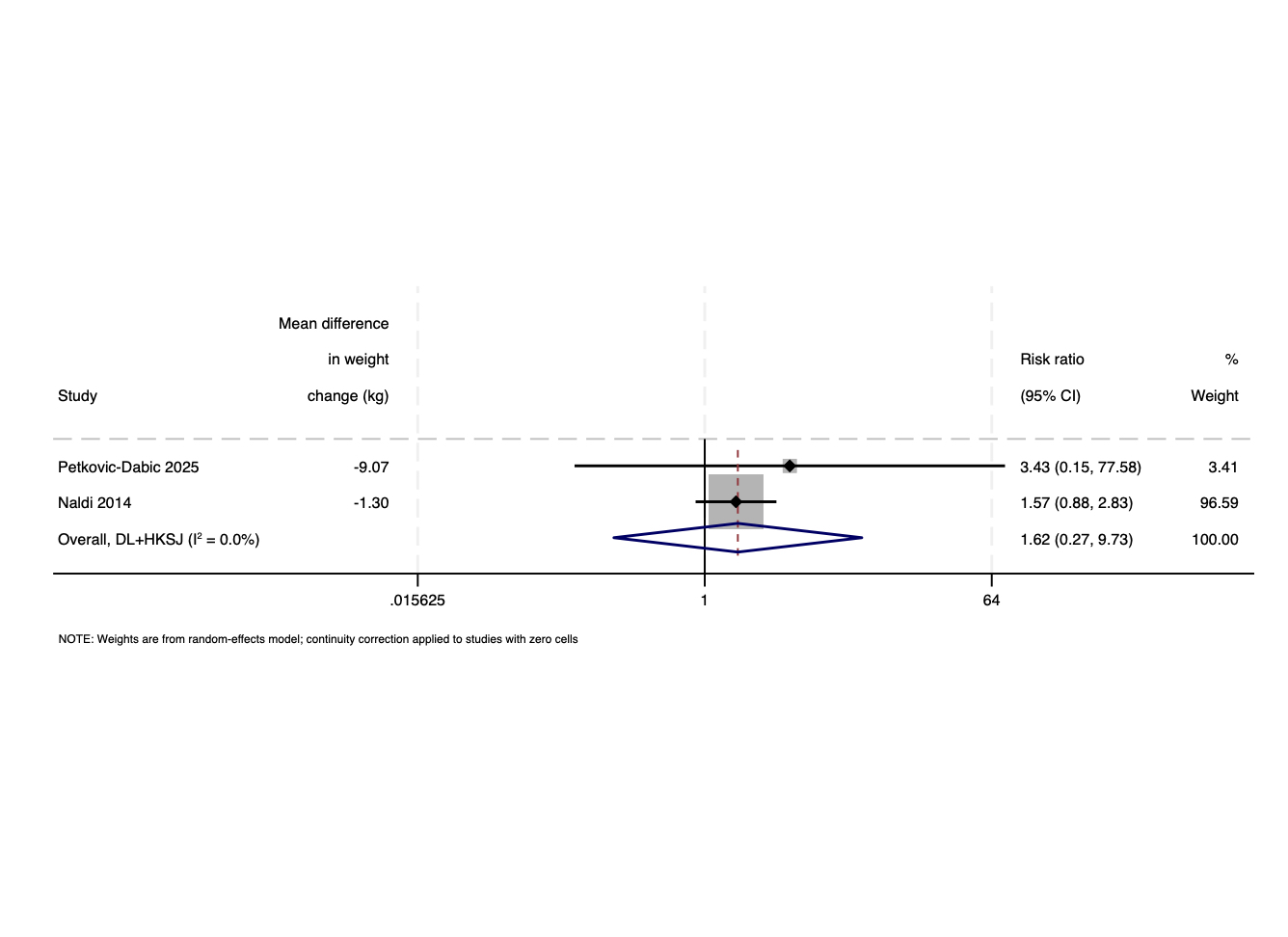
**

**FIGURE S8:** Sensitivity analysis. Forest plot to compare the change in DLQI score after a weight-loss intervention compared to control, using the random effects model, excluding studies which were judged to be at high risk of bias.

**
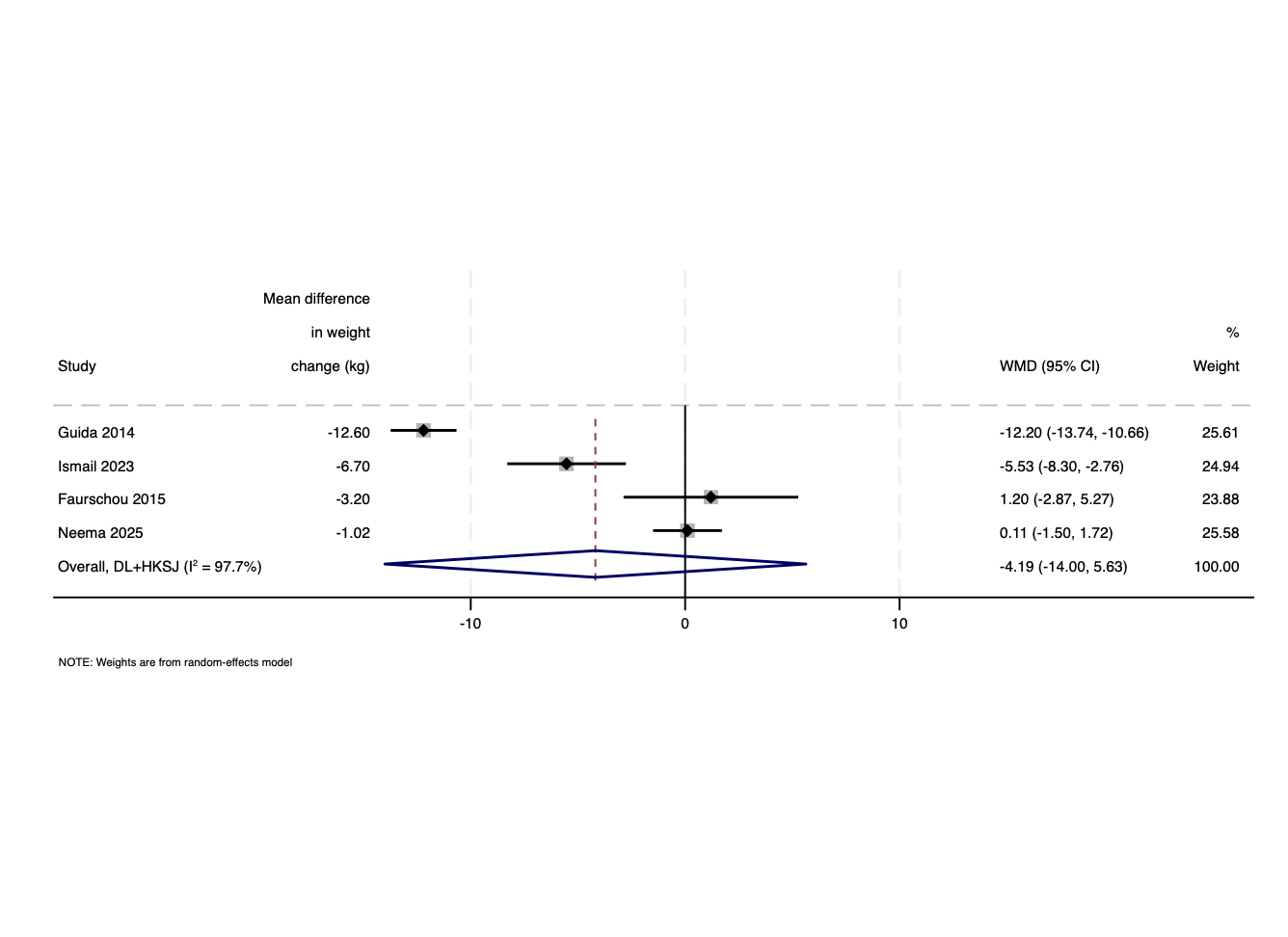
**

**FIGURE S9:** Sensitivity analysis. Forest plot to compare the change in PASI score after a weight-loss intervention compared to control, using random effects model, excluding studies where participant mean baseline PASI score was <5 (mild disease).


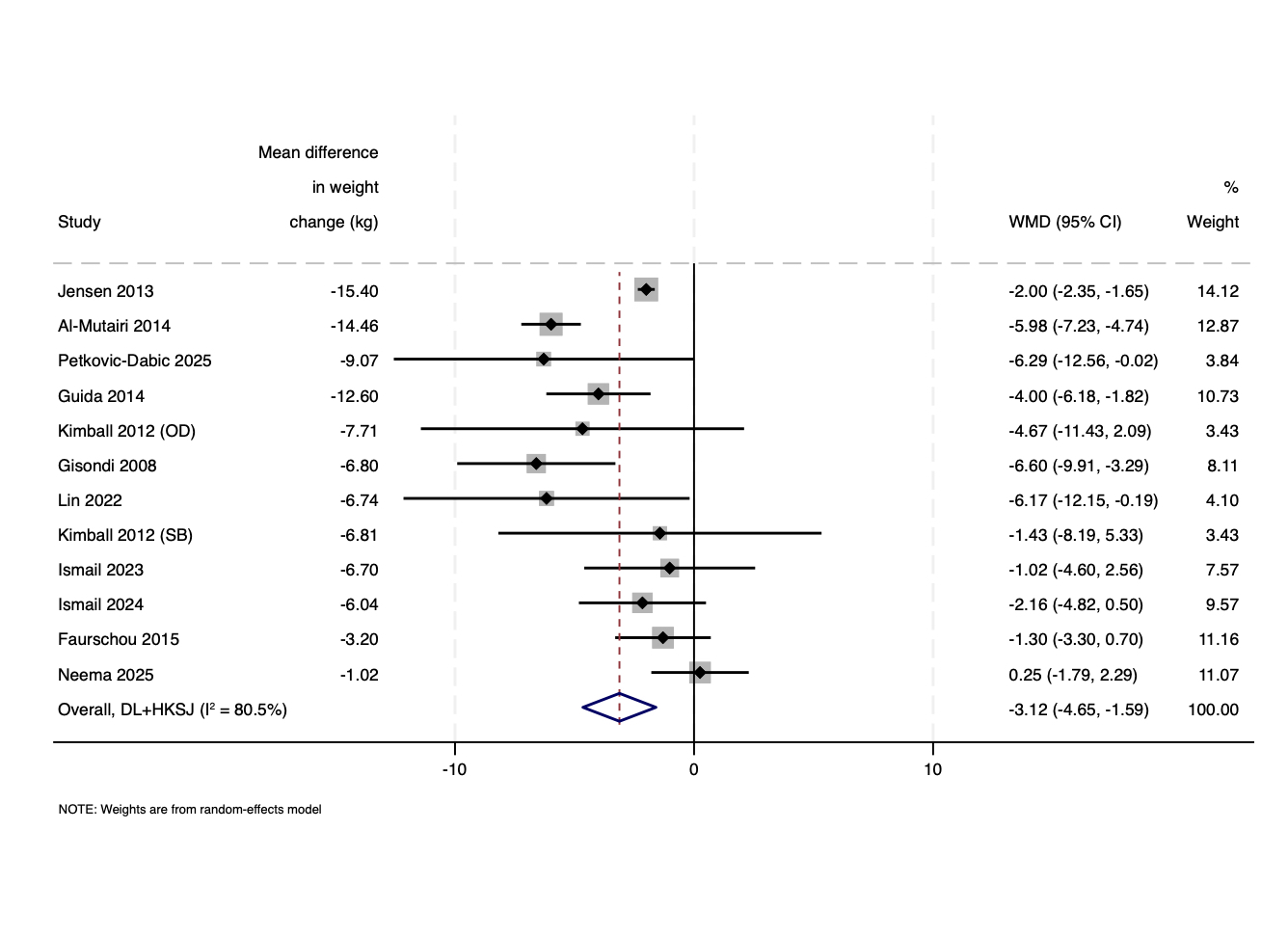


**FIGURE S10:** Sensitivity analysis. Forest plot to compare the change in PASI score after a weight-loss intervention compared to control, using the random effects model, excluding studies where weight-loss was minimally different between groups or greater in control group (<1.5kg difference in weight-loss between groups).


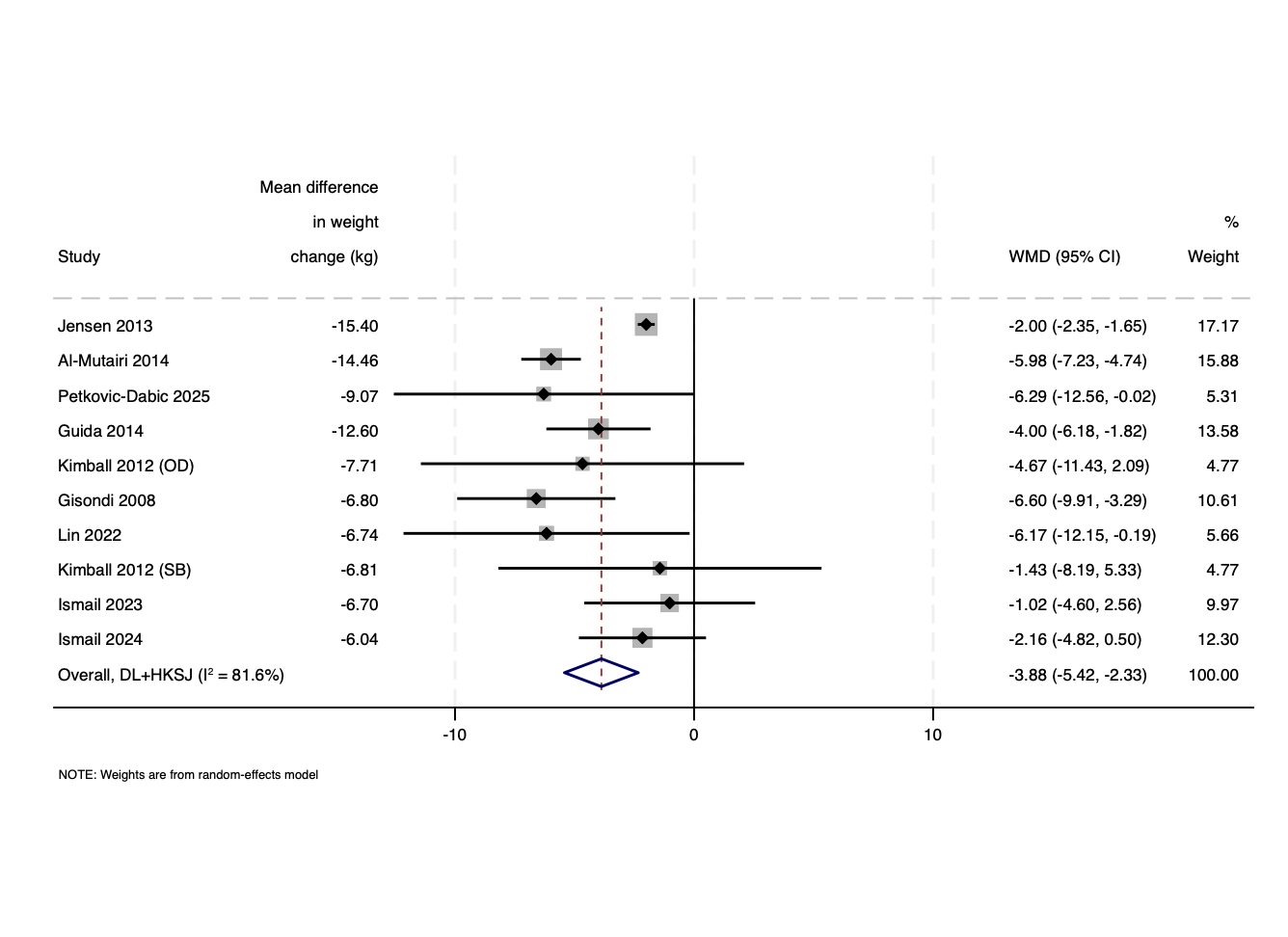


**FIGURE S11:** Sensitivity analysis. Forest plot to compare the change in PASI after a weight-loss intervention compared to control, using the random effects model, excluding one study where psoriasis medical treatments were different between intervention groups.

**
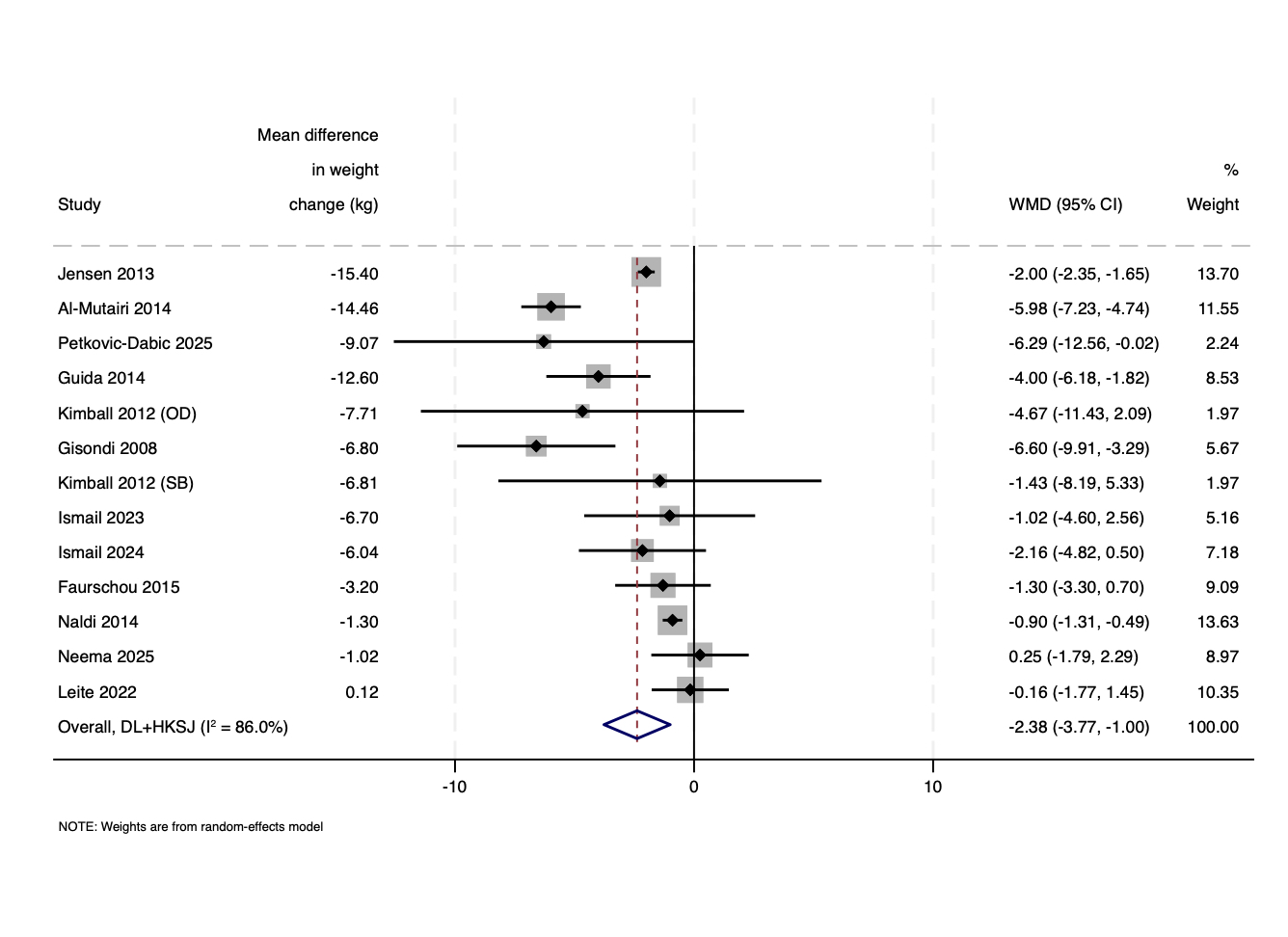
**

**FIGURE S12:** Sensitivity analysis. Forest plot demonstrating the risk of achieving PASI 75 in weight-loss intervention participants compared to control participants, excluding one study where psoriasis medical treatments were different between intervention groups.

**
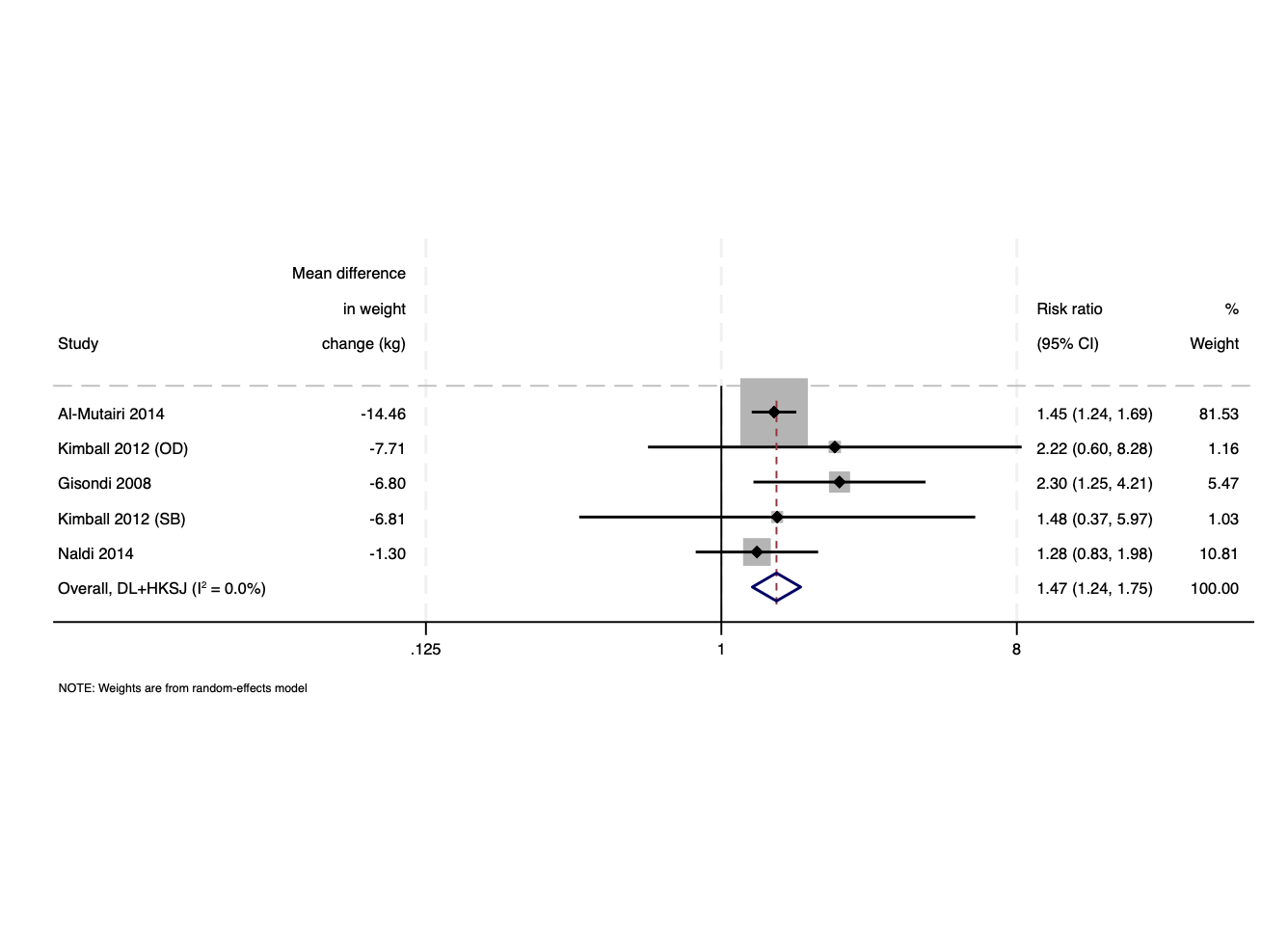
**

**FIGURE S13:** Sensitivity analysis. Forest plot demonstrating the risk of achieving PASI 50 in weight-loss intervention participants compared to control participants, excluding one study where psoriasis medical treatments were different between intervention groups.

**
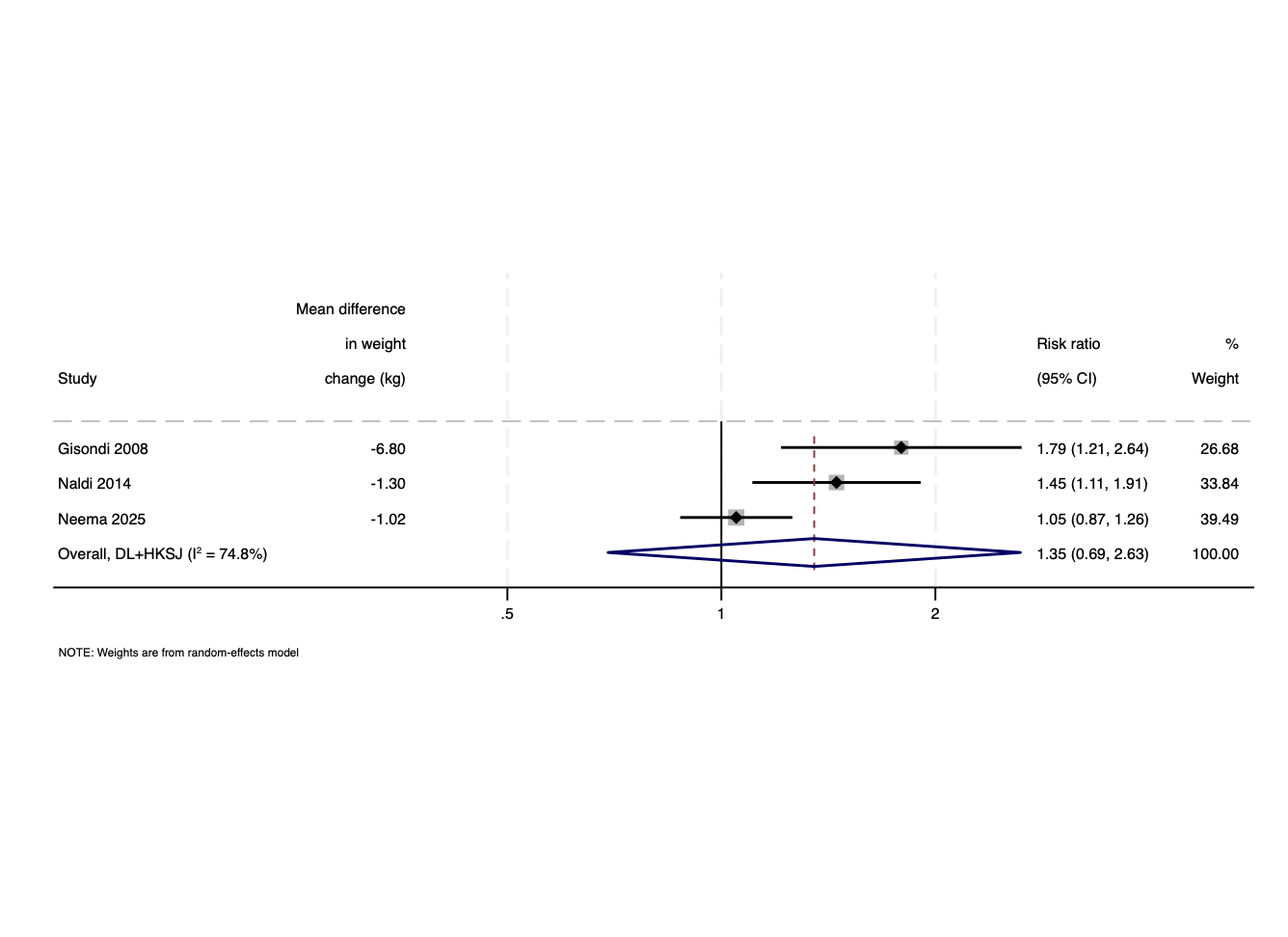
**

**FIGURE S14:** Sensitivity analysis. Forest plot to compare the change in DLQI score after a weight-loss intervention compared to control, using the random effects model, excluding one study where psoriasis medical treatments were different between intervention groups.

**
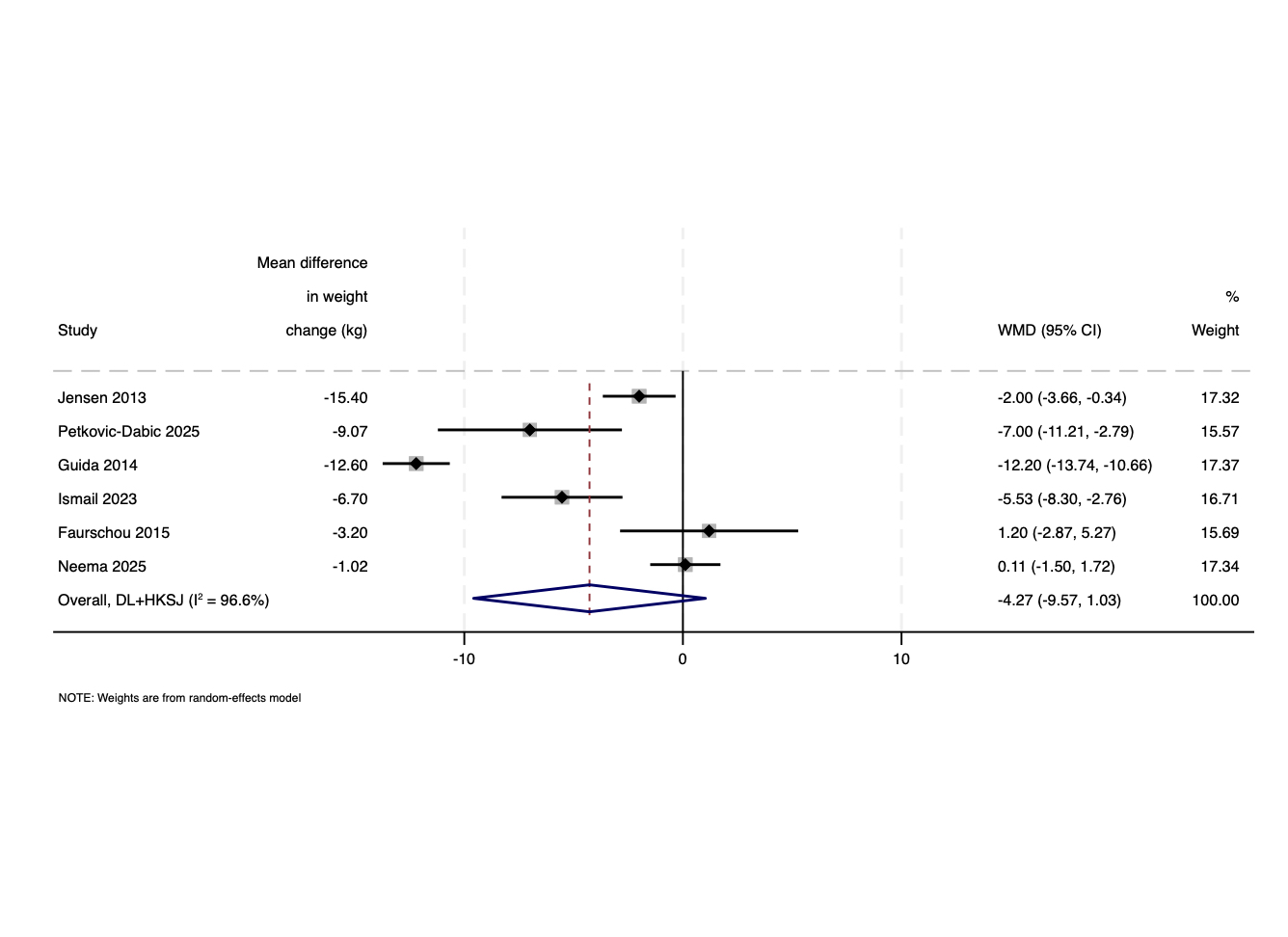
**

**FIGURE S15:** Subgroup analysis. Forest plot comparing mean difference in weight change (kg) between intervention groups vs control groups. Divided into three subgroups: diet alone (Subgroup 1), diet plus physical activity (Subgroup 2) and pharmacological weight-loss agent (Subgroup 3).


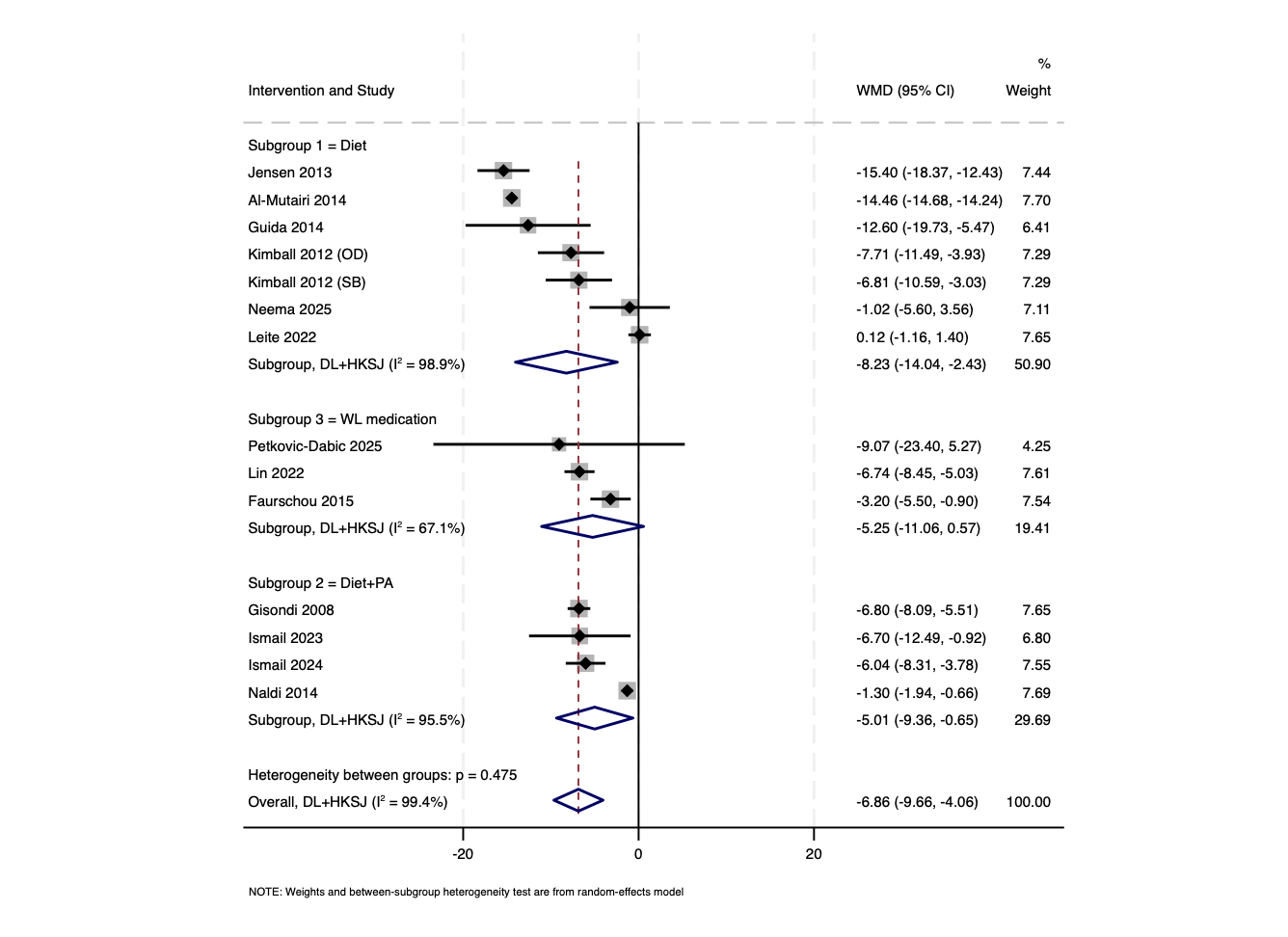


**FIGURE S16:** Subgroup analysis. Forest plot comparing PASI change between weight-loss intervention and control groups. Divided into three subgroups: diet alone (Subgroup 1), diet plus physical activity (Subgroup 2) and pharmacological weight-loss agent (Subgroup 3).


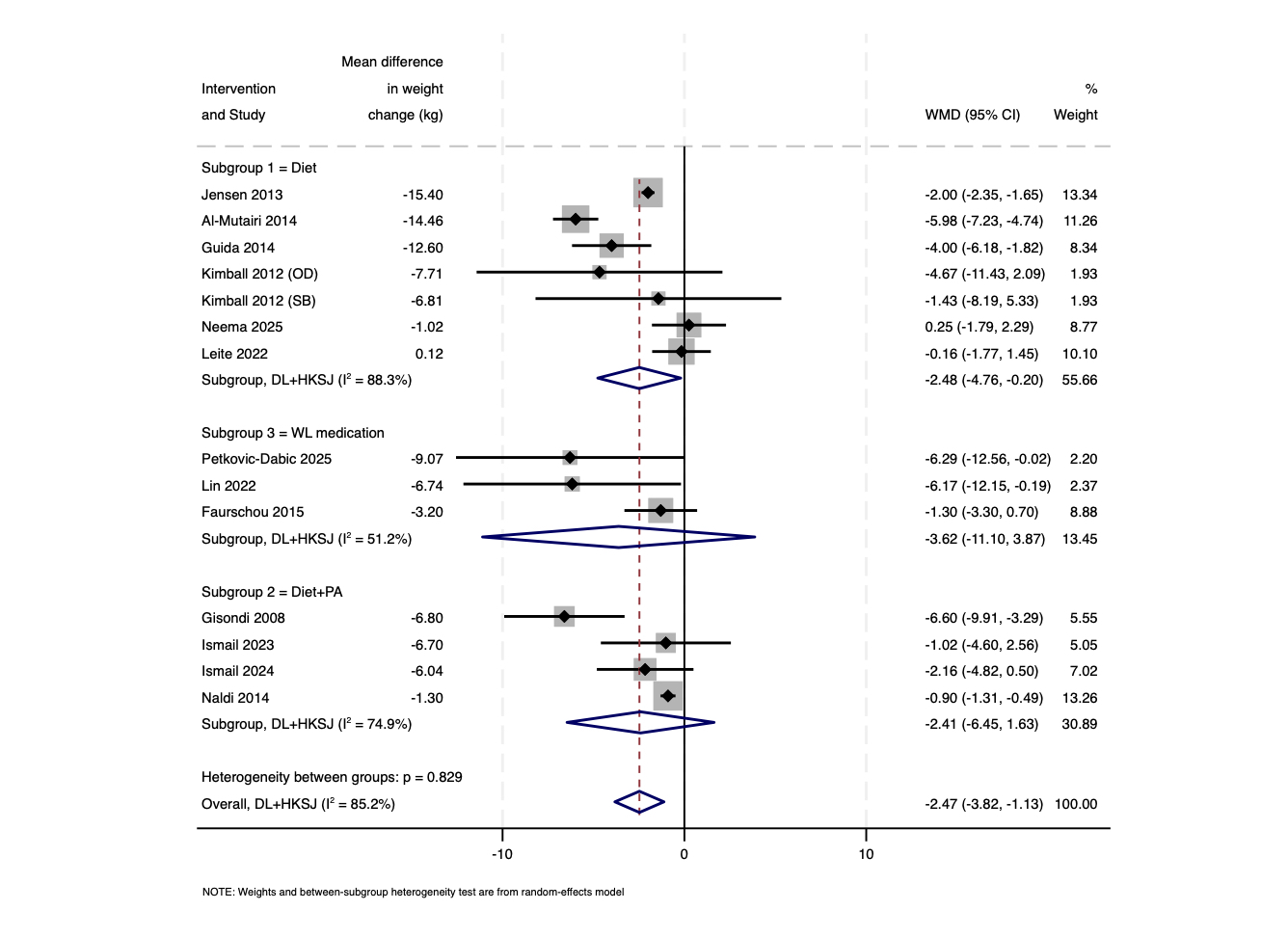


**FIGURE S17:** Subgroup analysis. Forest plot comparing PASI change between weight-loss intervention and control groups. Divided into two subgroups: ‘short’ duration of intervention (≤12 weeks) (Subgroup 1) and ‘medium’ duration (>12 weeks) (Subgroup 2).


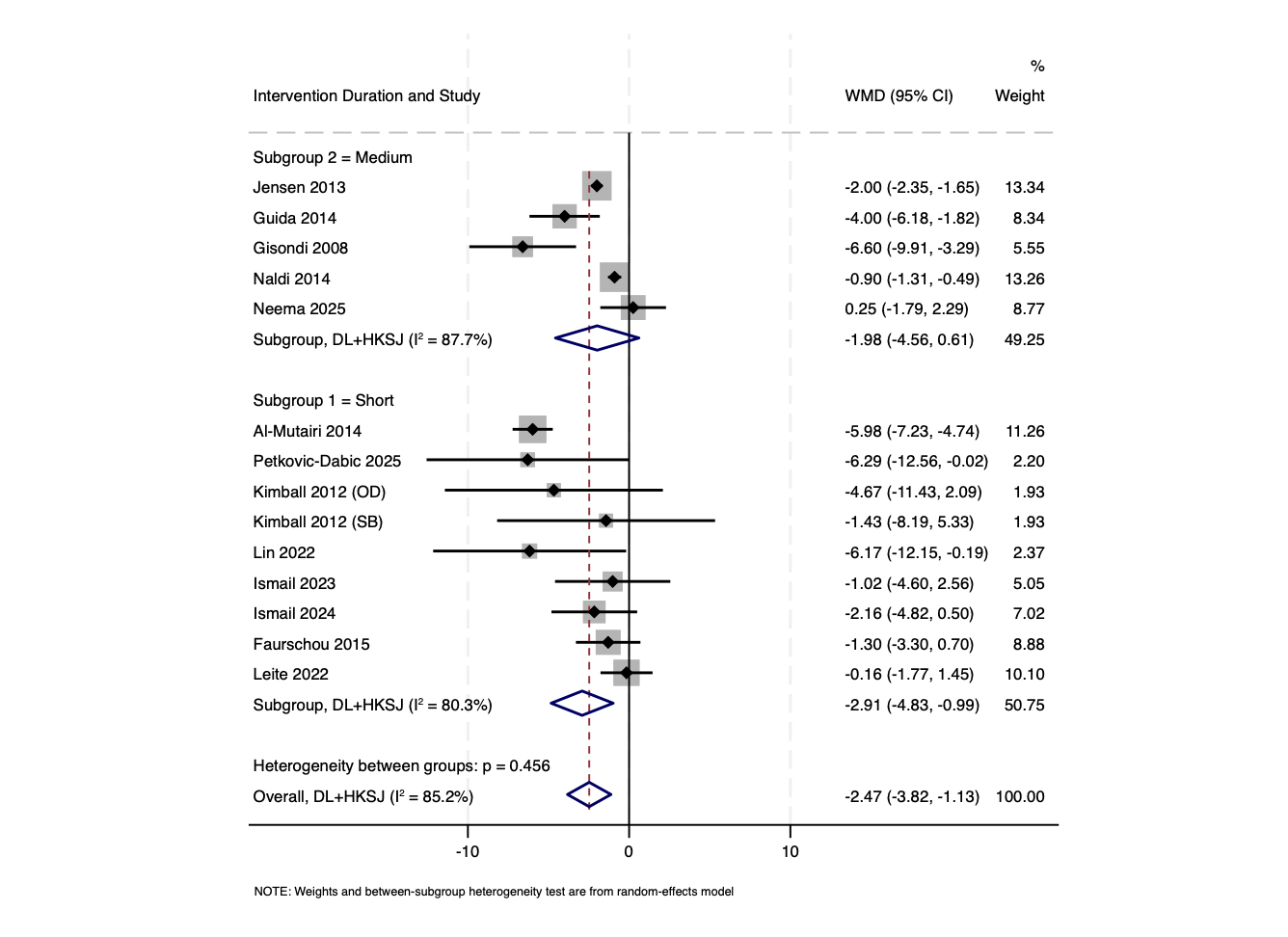


**FIGURE S18:** Subgroup analysis. Forest plot comparing PASI change between weight-loss intervention and control groups. Divided into three subgroups: Baseline PASI≤5 (Subgroup 1), Baseline PASI 5<PASI≤10 (Subgroup 2) and Baseline PASI>10 (Subgroup 3).


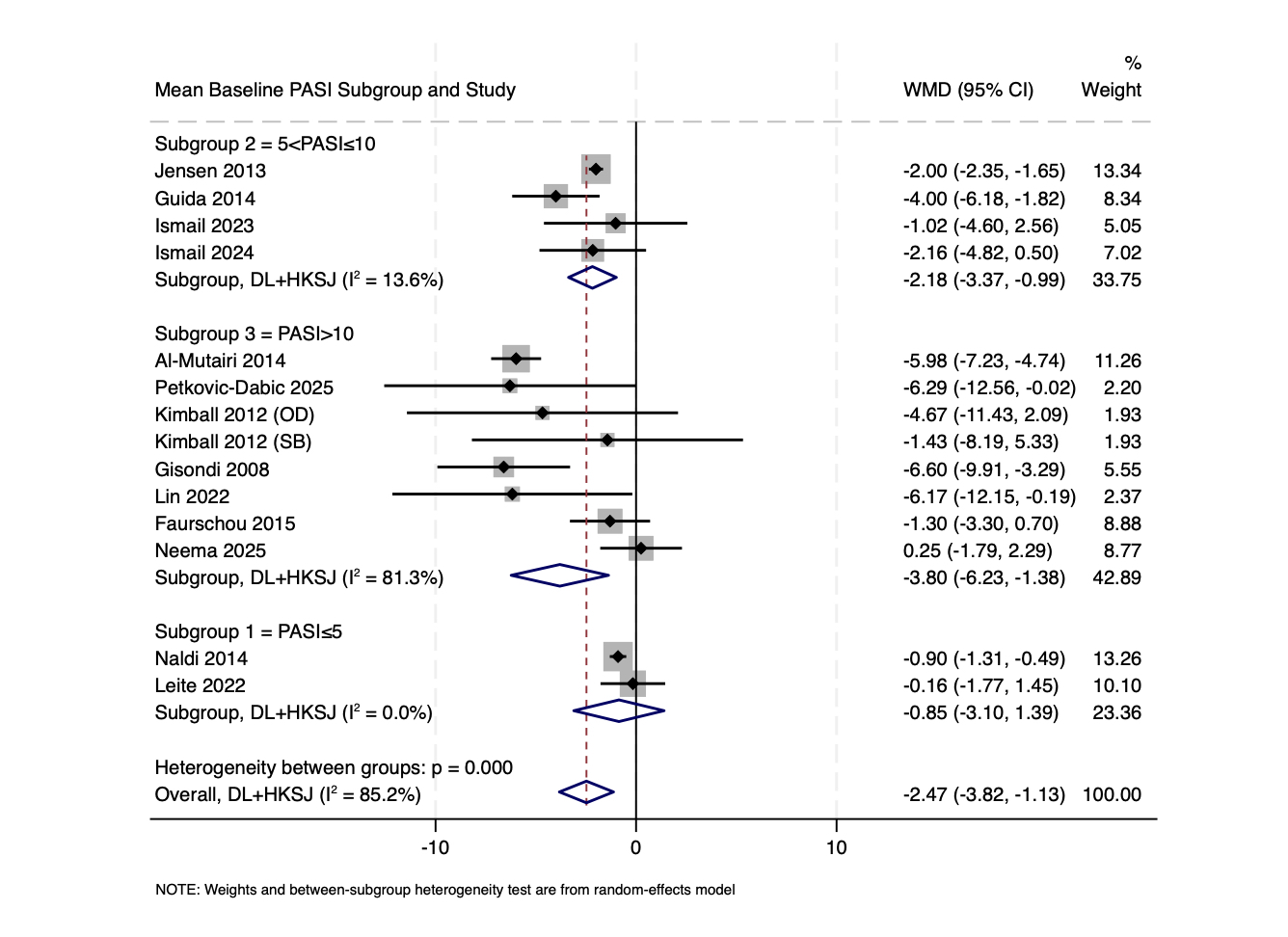

Supplement: Supplementary file 2 — Data S2. [file JDV-40-980-s002.docx]
