# Supplementary material for: Impact of weight‐loss interventions on psoriasis severity: A systematic review and meta‐analysis
Source: J Eur Acad Dermatol Venereol. 2025 Dec 19;40(6):980–93. doi: 10.1111/jdv.70247 (PMC13206337; doi:10.1111/jdv.70247)
Supplement: Supplementary file 3 — Appendix S1. [file JDV-40-980-s001.docx]

# Appendix S1: Search strategy

Eli Harriss (Bodleian Health Care Libraries, University of Oxford, ORCID: 0000-0003-4635-8959) created and translated the searches on 09/09/2024 and managed the results. References were exported to EndNote 20 (Thomson Reuters) and de-duplicated in Covidence.

**Database: Medline (Ovid MEDLINE® Epub Ahead of Print, In-Process & Other Non-Indexed Citations, Ovid MEDLINE® Daily and Ovid MEDLINE®) 1946 to present**

Link to search history: <https://ovidsp.ovid.com/ovidweb.cgi?T=JS&NEWS=N&PAGE=main&SHAREDSEARCHID=6m2myQk1XzaFJhzO7zt8I1sV7chg4CFr1Fv2D8Z6QxPoecToUIhXqz5ii9Ux7lB6q>
Search Strategy:
1  randomized controlled trial.pt. (604235)
2  controlled clinical trial.pt. (95474)
3  randomized.ab. (626900)
4  placebo.ab. (243680)
5  drug therapy.fs. (2644937)
6  randomly.ab. (422276)
7  trial.ab. (675554)
8  groups.ab. (2605458)
9  1 or 2 or 3 or 4 or 5 or 6 or 7 or 8 (5821034)
10  exp animals/ not humans.sh. (5175557)
11  9 not 10 (5086366)
12  exp Psoriasis/ (48788)
13  psoria*.ti,ab,kf. (61677)
14  12 or 13 (67782)
15  body mass index/ (150579)
16  BMI.ti,ab,kf. (199565)
17  "body mass index*".ti,ab,kf. (245168)
18  exp obesity/ (265434)
19  exp body weight/ (538238)
20  Adiposity/ (15671)
21  (obese or obesity or overweight or weight or adiposity or excess body fat).ti,ab,kf. (1278306)
22  Glycemic Index/ (3714)
23  Glycemic Load/ (368)
24  (low adj6 (glycemic index or glycaemic index or glycaemic load or glycemic load)).tw. (1643)
25  (diet? adj6 (glycemic index or glycaemic index or glycaemic load or glycemic load)).tw. (1146)
26  (food? adj6 (glycemic index or glycaemic index or glycaemic load or glycemic load)).tw. (842)
27  (diet? adj6 (low energy or low insulin or energy restricted or low carb*)).tw. (5084)
28  exp Anti-Obesity Agents/ (20674)
29  ("anti-obesity agent*" or "weight-loss agent*" or "anti obesity drug*" or "weight-loss drug*" or orlistat or tetrahydrolipstatin or empagliflozin or liraglutide or exenatide or exendin or lixisenatide or dulaglutide or weight reduction programs or "reducing diet*").ti,ab,kf. (15592)
30  Weight Reduction Programs/ (2840)
31  exp Exercise/ (250973)
32  (physical education and training).mp. [mp=title, book title, abstract, original title, name of substance word, subject heading word, floating sub-heading word, keyword heading word, organism supplementary concept word, protocol supplementary concept word, rare disease supplementary concept word, unique identifier, synonyms, population supplementary concept word, anatomy supplementary concept word] (14898)
33  "Physical Education and Training"/ (14234)
34  exp Physical Fitness/ (36689)
35  exp Life Style/ (111854)
36  exp Exercise Therapy/ (64415)
37  exp Diet Therapy/ (62944)
38  (exercis* or "physical* activ*" or lifestyle or "life style" or "diet* therap*").ti,ab,kf. (599695)
39  exp Bariatric Surgery/ (34216)
40  ((bariatric or obes*) adj5 surg*).ti,ab. or bariatric*.ti. (30685)
41  (((gastric or jejunoileal) adj3 (band* or bypass* or balloon* or diver*)) or gastrectom* or gastroplast* or ((biliopancreatic or bilio-pancreatic) adj2 diver*)).ti,ab. (53414)
42  obesity management/ or bariatrics/ (778)
43  15 or 16 or 17 or 18 or 19 or 20 or 21 or 22 or 23 or 24 or 25 or 26 or 27 or 28 or 29 or 30 or 31 or 32 or 33 or 34 or 35 or 36 or 37 or 38 or 39 or 40 or 41 or 42 (2308667)
44  11 and 14 and 43 (1521)

**Database: Embase 1974 to present**

Link to search history: <https://ovidsp.ovid.com/ovidweb.cgi?T=JS&NEWS=N&PAGE=main&SHAREDSEARCHID=5gyyZTJaQyzawvey9NvTEv54wjOa18lAMLu9fSPvy38OTWE3O3UPp3KhL2DwbqUbg>
Search Strategy:
1  randomized controlled trial/ (795567)
2  controlled clinical trial/ (471618)
3  random*.ti,ab. (2004049)
4  randomization/ (98914)
5  intermethod comparison/ (302941)
6  placebo.ti,ab. (369166)
7  (compare or compared or comparison).ti. (611357)
8  ((evaluated or evaluate or evaluating or assessed or assess) and (compare or compared or comparing or comparison)).ab. (2823541)
9  (open adj label).ti,ab. (111162)
10  ((double or single or doubly or singly) adj (blind or blinded or blindly)).ti,ab. (276647)
11  double blind procedure/ (213246)
12  parallel group*1.ti,ab. (32584)
13  (crossover or cross over).ti,ab. (125753)
14  ((assign$ or match or matched or allocation) adj5 (alternate or group$1 or intervention$1 or patient$1 or subject$1 or participant$1)).ti,ab. (420891)
15  (assigned or allocated).ti,ab. (497349)
16  (controlled adj7 (study or design or trial)).ti,ab. (457078)
17  (volunteer or volunteers).ti,ab. (284691)
18  human experiment/ (651006)
19  trial.ti. (408419)
20  1 or 2 or 3 or 4 or 5 or 6 or 7 or 8 or 9 or 10 or 11 or 12 or 13 or 14 or 15 or 16 or 17 or 18 or 19 (6412798)
21  (rat or rats or mouse or mice or swine or porcine or murine or sheep or lambs or pigs or piglets or rabbit or rabbits or cat or cats or dog or dogs or cattle or bovine or monkey or monkeys or trout or marmoset$1).ti. and animal experiment/ (1231881)
22  Animal experiment/ not (human experiment/ or human/) (2587600)
23  21 or 22 (2657506)
24  20 not 23 (6023184)
25  exp psoriasis/ (113215)
26  psoria*.ti,ab,kf. (96778)
27  25 or 26 (123391)
28  body mass/ (651280)
29  BMI.ti,ab,kf. (418141)
30  "body mass index*".ti,ab,kf. (358015)
31  exp obesity/ (675764)
32  exp body weight/ (666704)
33  (obese or obesity or overweight or weight or adiposity or excess body fat).ti,ab,kf. (1752465)
34  glycemic index/ (8410)
35  glycemic load/ (2005)
36  (low adj6 (glycemic index or glycaemic index or glycaemic load or glycemic load)).tw. (2395)
37  (diet? adj6 (glycemic index or glycaemic index or glycaemic load or glycemic load)).tw. (1731)
38  (food? adj6 (glycemic index or glycaemic index or glycaemic load or glycemic load)).tw. (1179)
39  (diet? adj6 (low energy or low insulin or energy restricted or low carb*)).tw. (6934)
40  exp antiobesity agent/ (22378)
41  orlistat.mp. or exp tetrahydrolipstatin/ (7915)
42  empagliflozin.mp. or exp empagliflozin/ (9151)
43  exp liraglutide/ or liraglutide.mp. (13640)
44  exenatide.mp. or exp exendin 4/ (12710)
45  exp lixisenatide/ or lixisenatide.mp. (2407)
46  dulaglutide.mp. or exp dulaglutide/ (3034)
47  weight reduction program.mp. or exp weight-loss program/ (3869)
48  reducing diet.mp. or exp low calory diet/ (2777)
49  ("anti-obesity agent*" or "weight-loss agent*" or "anti obesity drug*" or "weight-loss drug*" or tetrahydrolipstatin or exendin).ti,ab,kf. (6477)
50  exp exercise/ (439022)
51  (physical education and training).mp. (5222)
52  exp kinesiotherapy/ (101470)
53  fitness/ (42947)
54  exp lifestyle/ (167457)
55  exp diet therapy/ (420284)
56  (exercis* or "physical* activ*" or lifestyle or "life style" or "diet* therap*").ti,ab,kf. (803967)
57  exp bariatric surgery/ (67591)
58  ((weight-loss or bariatric or obes*) adj5 surg*).ti,ab. or bariatric*.ti. (57304)
59  (((gastric or jejunoileal) adj3 (band* or bypass* or balloon* or diver*)) or gastrectom* or gastroplast* or ((biliopancreatic or bilio-pancreatic) adj2 diver*)).ti,ab. (79679)
60  obesity management/ or bariatrics/ (2169)
61  (obes* adj3 manage*).ti,ab. (8610)
62  28 or 29 or 30 or 31 or 32 or 33 or 34 or 35 or 36 or 37 or 38 or 39 or 40 or 41 or 42 or 43 or 44 or 45 or 46 or 47 or 48 or 49 or 50 or 51 or 52 or 53 or 54 or 55 or 56 or 57 or 58 or 59 or 60 or 61 (3575562)
63  24 and 27 and 62 (3761)

**Database: PsycINFO 1806 to present**

Link to search history: <https://ovidsp.ovid.com/ovidweb.cgi?T=JS&NEWS=N&PAGE=main&SHAREDSEARCHID=6m2myQk1XzaFJhzO7zt8I1xCJECcx7uFB13F55PRUKJnZN38qYoqGA4e5lRkPZ9PS>
Search Strategy:
1  (random* or trial* or controlled stud* or placebo* or ((singl* or doubl* or trebl* or tripl*) and (blind* or mask*)) or cross over or crossover or factorial* or latin square or assign* or allocat* or volunteer*).ti,ab,hw,id. or treatment effectiveness evaluation/ or mental health program evaluation/ or exp experimental design/ or (clinical trial or treatment outcome).md. (623403)
2  psoria*.mp. (821)
3  (BMI or "body mass index" or obes* or overweight or weight* or adiposity or excess body fat).mp. (179365)
4  (low adj6 (glycemic index or glycaemic index or glycaemic load or glycemic load)).mp. (88)
5  (diet? adj6 (glycemic index or glycaemic index or glycaemic load or glycemic load)).mp. (68)
6  (food? adj6 (glycemic index or glycaemic index or glycaemic load or glycemic load)).mp. (44)
7  (diet? adj6 (low energy or low insulin or energy restricted or low carb*)).mp. (319)
8  ("anti-obesity agent*" or "weight-loss agent*" or "anti obesity drug*" or "weight-loss drug*" or orlistat or tetrahydrolipstatin or empagliflozin or liraglutide or exenatide or exendin or lixisenatide or dulaglutide or weight reduction programs or "reducing diet*").mp. (1414)
9  (exercis* or "physical* activ*" or lifestyle or "life style" or "diet* therap*").mp. (156968)
10  bariatric*.mp. (1906)
11  (((gastric or jejunoileal) adj3 (band* or bypass* or balloon* or diver*)) or gastrectom* or gastroplast* or ((biliopancreatic or bilio-pancreatic) adj2 diver*)).mp. (842)
12  3 or 4 or 5 or 6 or 7 or 8 or 9 or 10 or 11 (307989)
13  1 and 2 and 12 (18)

Top of Form

| **EBSCOhost CINAHL**  **#** | **Query** | **Results** |
| --- | --- | --- |
| S1 | MH randomized controlled trials | 140,193 |
| S2 | MH double-blind studies | 54,334 |
| S3 | MH single-blind studies | 16,072 |
| S4 | MH random assignment | 82,209 |
| S5 | MH pretest-posttest design | 54,224 |
| S6 | MH cluster sample | 5,358 |
| S7 | TI (randomised OR randomized) | 145,379 |
| S8 | AB (random*) | 403,213 |
| S9 | TI (trial) | 186,567 |
| S10 | MH (sample size) AND AB (assigned OR allocated OR control) | 4,452 |
| S11 | MH (placebos) | 13,881 |
| S12 | PT (randomized controlled trial) | 153,953 |
| S13 | AB (control W5 group) | 146,598 |
| S14 | MH (crossover design) OR MH (comparative studies) | 482,885 |
| S15 | AB (cluster W3 RCT) | 503 |
| S16 | MH animals+ | 103,096 |
| S17 | MH (animal studies) | 154,598 |
| S18 | TI (animal model*) | 3,814 |
| S19 | S16 OR S17 OR S18 | 248,740 |
| S20 | MH (human) | 2,731,992 |
| S21 | S19 NOT S20 | 214,616 |
| S22 | S1 OR S2 OR S3 OR S4 OR S5 OR S6 OR S7 OR S8 OR S9 OR S10 OR S11 OR S12 OR S13 OR S14 OR S15 | 1,029,514 |
| S23 | S22 NOT S21 | 981,578 |
| S24 | (MH "Psoriasis+") | 9,424 |
| S25 | TI psoria* OR AB psoria* | 12,145 |
| S26 | S24 OR S25 | 14,133 |
| S27 | (MH "Body Mass Index") | 96,670 |
| S28 | TI ( BMI or "body mass index" ) OR AB ( BMI or "body mass index" ) | 113,361 |
| S29 | (MH "Obesity+") | 114,979 |
| S30 | (MH "Body Weight+") | 174,665 |
| S31 | TI ( obese or obesity or overweight or weight or adiposity or "excess body fat" ) OR AB ( obese or obesity or overweight or weight or adiposity or "excess body fat" ) | 282,919 |
| S32 | (MH "Glycemic Index") | 2,999 |
| S33 | (MH "Glycemic Load") | 447 |
| S34 | TI ( low n6 (glycemic index or glycaemic index or glycaemic load or glycemic load) ) OR AB ( low n6 (glycemic index or glycaemic index or glycaemic load or glycemic load) ) | 824 |
| S35 | TI ( diet* n6 (glycemic index or glycaemic index or glycaemic load or glycemic load) ) OR AB ( diet* n6 (glycemic index or glycaemic index or glycaemic load or glycemic load) ) | 1,144 |
| S36 | TI ( food* n6 (glycemic index or glycaemic index or glycaemic load or glycemic load) ) OR AB ( food* n6 (glycemic index or glycaemic index or glycaemic load or glycemic load) ) | 428 |
| S37 | TI ( diet* n6 (low energy or low insulin or energy restricted or low carb*) ) OR AB ( diet* n6 (low energy or low insulin or energy restricted or low carb*) ) | 2,802 |
| S38 | (MH "Antiobesity Agents+") | 7,998 |
| S39 | TI ( ("anti-obesity agent*" or "weight-loss agent*" or "anti obesity drug*" or "weight-loss drug*" or orlistat or tetrahydrolipstatin or empagliflozin or liraglutide or exenatide or exendin or lixisenatide or dulaglutide or weight reduction programs or "reducing diet*") ) OR AB ( ("anti-obesity agent*" or "weight-loss agent*" or "anti obesity drug*" or "weight-loss drug*" or orlistat or tetrahydrolipstatin or empagliflozin or liraglutide or exenatide or exendin or lixisenatide or dulaglutide or weight reduction programs or "reducing diet*") ) | 4,417 |
| S40 | (MH "Weight Reduction Programs") | 3,388 |
| S41 | (MH "Exercise+") | 129,464 |
| S42 | (MH "Physical Education and Training+") | 4,716 |
| S43 | (MH "Physical Fitness+") | 21,202 |
| S44 | (MH "Life Style+") | 278,600 |
| S45 | (MH "Therapeutic Exercise+") | 63,608 |
| S46 | (MH "Diet Therapy+") | 36,269 |
| S47 | TI ( exercis* or "physical* activ*" or lifestyle or "life style" or "diet* therap*" ) OR AB ( exercis* or "physical* activ*" or lifestyle or "life style" or "diet* therap*" ) | 252,610 |
| S48 | (MH "Bariatric Surgery+") | 9,784 |
| S49 | TI bariatric* OR AB bariatric* | 8,408 |
| S50 | TI ( (((gastric or jejunoileal) n3 (band* or bypass* or balloon* or diver*)) or gastrectom* or gastroplast* or ((biliopancreatic or bilio-pancreatic) n2 diver*)) ) OR AB ( (((gastric or jejunoileal) n3 (band* or bypass* or balloon* or diver*)) or gastrectom* or gastroplast* or ((biliopancreatic or bilio-pancreatic) n2 diver*)) ) | 8,834 |
| S51 | S27 OR S28 OR S29 OR S30 OR S31 OR S32 OR S33 OR S34 OR S35 OR S36 OR S37 OR S38 OR S39 OR S40 OR S41 OR S42 OR S43 OR S44 OR S45 OR S46 OR S47 OR S48 OR S49 OR S50 | 914,102 |
| S52 | S23 AND S26 AND S51 | 254 |

Bottom of Form

**Cochrane Central Register of Controlled Trials**

**Issue 11 of 12, November 2023**

#1 psoria*:ti,ab,kw 11310

#2 (bmi OR (body near mass near index)):ti,ab,kw 85752

#3 (obese or obesity or overweight or weight or adiposity or (excess body fat)):ti,ab,kw 168015

#4 (low near/6 (glycemic index or glycaemic index or glycaemic load or glycemic load)):ti,ab,kw 4927

#5 (diet* near/6 (glycemic index or glycaemic index or glycaemic load or glycemic load)):ti,ab,kw 4052

#6 (food* near/6 (glycemic index or glycaemic index or glycaemic load or glycemic load)):ti,ab,kw 1454

#7 (diet* near/6 (low energy or low insulin or energy restricted or low carb*)):ti,ab,kw 23571

#8 (anti-obesity agent* or weight-loss agent* or anti obesity drug* or weight-loss drug* or orlistat or tetrahydrolipstatin or empagliflozin or liraglutide or exenatide or exendin or lixisenatide or dulaglutide or weight reduction programs or reducing diet*):ti,ab,kw 28476

#9 (exercis* or physical* activ* or lifestyle or life style or diet therap* or dietary therap*):ti,ab,kw 228832

#10 ((bariatric or obes*) near/5 surg*):ti,ab,kw 4830

#11 bariatric*:ti 1681

#12 (((gastric or jejunoileal) near/3 (band* or bypass* or balloon* or diver*)) or gastrectom* or gastroplast* or ((biliopancreatic or bilio-pancreatic) near/2 diver*)):ti,ab,kw 7065

#13 #2 or #3 or #4 or #5 or #6 or #7 or #8 or #9 or #10 or #11 or #12 390089

#14 #1 and #13 1610

**Web of Science – Core Collection**

#1 **random* or blind* or allocat* or assign* or trial* or placebo* or crossover* or cross-over*** (Topic) not **animals or  animal  or  mice  or  mus  or  mouse  or  murine  or  woodmouse  or  rats  or  rat  or  murinae  or  muridae  or  cottonrat  or  cottonrats  or  hamster  or  hamsters  or  cricetinae  or  rodentia  or  rodent  or  rodents  or  pigs  or  pig  or  swine  or  swines  or  piglets  or  piglet  or  boar  or  boars  or  sus  scrofa  or  ferrets  or  ferret  or  polecat  or  polecats  or  mustela  putorius  or  guinea  pigs  or  guinea  pig  or  cavia  or  callithrix  or  marmoset  or  marmosets  or  cebuella  or  hapale  or  octodon  or  chinchilla  or  chinchillas  or  gerbillinae  or  gerbil  or  gerbils  or  jird  or  jirds  or  merione  or  meriones  or  rabbits  or  rabbit  or  hares  or  hare  or  diptera  or  flies  or  fly  or  dipteral  or  drosphila  or  drosophilidae  or  cats  or  cat  or  carus  or  felis  or  nematoda  or  nematode  or  nematoda  or  nematode  or  nematodes  or  sipunculida  or  dogs  or  dog  or  canine  or  canines  or  canis  or  sheep  or  sheeps  or  mouflon  or  mouflons  or  ovis  or  goats  or  goat  or  capra  or  capras  or  rupicapra  or  chamois  or  haplorhini  or  monkey  or  monkeys  or  anthropoidea  or  anthropoids  or  saguinus  or  tamarin  or  tamarins  or  leontopithecus  or  hominidae  or  ape  or  apes  or  pan  or  paniscus  or  pan  paniscus  or  bonobo  or  bonobos  or  troglodytes  or  pan  troglodytes  or  gibbon  or  gibbons  or  siamang  or  siamangs  or  nomascus  or  symphalangus  or  chimpanzee  or  chimpanzees  or  prosimians  or  bush  baby  or  prosimian  or  bush  babies  or  galagos  or  galago  or  pongidae  or  gorilla  or  gorillas  or  pongo  or  pygmaeus  or  pongo  pygmaeus  or  orangutans  or  pygmaeus  or  lemur  or  lemurs  or  lemuridae  or  horse  or  horses  or  pongo  or  equus  or  cow  or  calf  or  bull  or  chicken  or  chickens  or  gallus  or  quail  or  bird  or  birds  or  quails  or  poultry  or  poultries  or  fowl  or  fowls  or  reptile  or  reptilia  or  reptiles  or  snakes  or  snake  or  lizard  or  lizards  or  alligator  or  alligators  or  crocodile  or  crocodiles  or  turtle  or  turtles  or  amphibian  or  amphibians  or  amphibia  or  frog  or  frogs  or  bombina  or  salientia  or  toad  or  toads  or  epidalea  calamita  or  salamander  or  salamanders  or  eel  or  eels  or  fish  or  fishes  or  pisces  or  catfish  or  catfishes  or  siluriformes  or  arius  or  heteropneustes  or  sheatfish  or  perch  or  perches  or  percidae  or  perca  or  trout  or  trouts  or  char  or  chars  or  salvelinus  or  fathead  minnow  or  minnow  or  cyprinidae  or  carps  or  carp  or  zebrafish  or  zebrafishes  or  goldfish  or  goldfishes  or  guppy  or  guppies  or  chub  or  chubs  or  tinca  or  barbels  or  barbus  or  pimephales  or  promelas  or  poecilia  reticulata  or  mullet  or  mullets  or  seahorse  or  seahorses  or  mugil  curema  or  atlantic  cod  or  shark  or  sharks  or  catshark  or  anguilla  or  salmonid  or  salmonids  or  whitefish  or  whitefishes  or  salmon  or  salmons  or  sole  or  solea  or  sea  lamprey  or  lamprey  or  lampreys  or  pumpkinseed  or  sunfish  or  sunfishes  or  tilapia  or  tilapias  or  turbot  or  turbots  or  flatfish  or  flatfishes  or  sciuridae  or  squirrel  or  squirrels  or  chipmunk  or  chipmunks  or  suslik  or  susliks  or  vole  or  voles  or  lemming  or  lemmings  or  muskrat  or  muskrats  or  lemmus  or  otter  or  otters  or  marten  or  martens  or  martes  or  weasel  or  badger  or  badgers  or  ermine  or  mink  or  minks  or  sable  or  sables  or  gulo  or  gulos  or  wolverine  or  wolverines  or  minks  or  mustela  or  llama  or  llamas  or  alpaca  or  alpacas  or  camelid  or  camelids  or  guanaco  or  guanacos  or  chiroptera  or  chiropteras  or  bat  or  bats  or  fox  or  foxes  or  iguana  or  iguanas  or  xenopus  laevis  or  parakeet  or  parakeets  or  parrot  or  parrots  or  donkey  or  donkeys  or  mule  or  mules  or  zebra  or  zebras  or  shrew  or  shrews  or  bison  or  bisons  or  buffalo  or  buffaloes  or  deer  or  deers  or  bear  or  bears  or  panda  or  pandas  or  wild  hog  or  wild  boar  or  fitchew  or  fitch  or  beaver  or  beavers  or  jerboa  or  jerboas  or  capybara  or  capybaras** (Topic)

#2 **BMI or "body mass index"** (Topic) or **obese or obesity or overweight or weight or adiposity or "excess body fat"** (Topic) or **low NEAR/3 ("glycemic index" or "glycaemic index" or "glycaemic load" or "glycemic load")** (Topic) or **diet* NEAR/3 ("glycemic index" or "glycaemic index" or "glycaemic load" or "glycemic load")** (Topic) or **food* NEAR/3 ("glycemic index" or "glycaemic index" or "glycaemic load" or "glycemic load")** (Topic) or **diet* NEAR/3 ("low energy" or "low insulin" or "energy restricted" or "low carb*")** (Topic)

#3 **"anti-obesity agent*" or "weight-loss agent*" or "anti obesity drug*" or "weight-loss drug*" or orlistat or tetrahydrolipstatin or empagliflozin or liraglutide or exenatide or exendin or lixisenatide or dulaglutide or weight reduction programs or "reducing diet*"** (Topic) or **exercis* or "physical* activ*" or lifestyle or "life style" or "diet* therap*"** (Topic) or **(bariatric or obes*) near/5 surg*** (Topic) or **(((gastric or jejunoileal) near/3 (band* or bypass* or balloon* or diver*)) or gastrectom* or gastroplast* or ((biliopancreatic or bilio-pancreatic) near/2 diver*))** (Topic)

#4 #2 or #3

#5 **TS=(psoria*)**

#6 #1 AND #4 AND #5

**Clinicaltrials.gov**

Condition or disease: Psoriasis

Other terms: weight

Condition or disease: Psoriasis

Other terms: obesity
